# Histone modification dynamics in brain aging: unlocking therapeutic potential

**DOI:** 10.1038/s41419-026-08917-5

**Published:** 2026-05-29

**Authors:** Bohao Chang, Sibo Yang, Aikedan Yilixiati, Feng Zhang, Xiaodi Sun, Luojinyun Wang, Bo Hu, Yifan Zhou

**Affiliations:** Department of Neurology, Union Hospital, Tongji Medical College, Huazhong University of Science and Technology, Wuhan, China

**Keywords:** Alzheimer's disease, Epigenetics and behaviour

## Abstract

During aging, the progressive decline in neuronal function contributes to cognitive impairment and predisposes individuals to neurodegenerative disorders. This phenomenon has become increasingly prominent in modern society. Recent studies have found that post-translational modifications (PTMs) of proteins play a crucial role in the aging process, influencing the physiological functions and pathological changes in brain cells. This article reviews the variety and complexity of PTMs across brain cell types during aging, analyzes changes in specific modification types and sites, and reveals their impacts on neurophysiological and pathological states. This review also examines the current state of translational research, noting that most of the drugs under investigation lack specificity, and their potential toxic side effects remain undetermined. In light of these concerns, this review proposes therapeutic strategies to guide innovative interventions for aging-related diseases, aiming to facilitate further research and clinical applications in this field.

## Highlights

- Histone modification is an important mechanism of brain cell aging.
- Histone modification controls the progression of neurodegenerative diseases.
- Understand the new therapeutic approaches of histone modification in brain aging and neurodegenerative diseases.

## Introduction

### Functional and cellular hallmarks of the aging brain

The profound implications of aging for human health and society make it paramount to understand its cellular and molecular basis. Aging constitutes a complex biological process characterized by significant physiological and pathological alterations. These changes drive functional decline and elevate mortality risk, with particular vulnerability observed in the central nervous system [1]. The aging brain undergoes structural alterations, most notably volumetric atrophy due to tissue loss [2]. These changes manifest clinically as a decline in cognitive functions, including learning, memory, attention, and decision-making, as well as disrupted sensory processing and reduced motor coordination [3]. Consistent with observations in other tissues, the aging brain exhibits a significant increase in cells expressing established senescence biomarkers, indicating that brain aging involves the progressive accumulation of senescent cells [4]. Sustained secretion of senescence-associated secretory phenotype (SASP) factors drives chronic inflammation (inflammaging) and establishes a disease-permissive microenvironment associated with aging-related dysfunction. Changes in synaptic plasticity in aging neurons disrupt normal neuronal network connections, as evidenced by the reduction in synapse number and abnormal synaptic transmission in several brain regions with aging [5]. Accounting for approximately 50% of brain cells, glia critically maintain brain homeostasis in health and disease through fundamental roles including neuronal metabolic support, immune response initiation, and regulation of synaptic transmission and plasticity [6]. The aging of glial cells increases neuroinflammation levels, leading to alterations in neuronal and synaptic function [7].

As the core of the nervous system, the brain is particularly vulnerable to aging-related degenerative diseases due to the limited regenerative capacity of neurons. Similarly, microglial aging leads to a substantial increase in immune-related issues within the brain [5]. Beyond the brain, the hallmarks of cellular aging are universally interconnected: telomere attrition, mitochondrial dysfunction with attendant oxidative stress, DNA repair impairment driving genomic instability, proteostatic collapse promoting protein misfolding, and epigenetic dysregulation.

Evidence suggests that aberrant epigenetic modifications may occur prior to events such as DNA damage and protein homeostasis imbalance, accelerating the aging process by silencing key genes, such as those involved in DNA repair. Shaped by diverse intrinsic and extrinsic factors over time, cumulative epigenetic modifications establish a distinct aging-associated landscape [8]. These alterations in epigenetic marks drive changes in gene expression, contributing to phenotypes associated with aging and the development of neurodegenerative diseases, including Alzheimer’s disease (AD), Parkinson’s disease (PD), and other degenerative disorders. Furthermore, neurodegenerative diseases such as AD exacerbate brain aging, with cross-sectional evidence revealing accelerated molecular aging pathways and pronounced structural deterioration in affected individuals compared with cognitively intact elders [9]. Nevertheless, the reversibility of epigenetic modifications by regulatory factors provides a mechanistic basis for aging intervention, positioning them as promising therapeutic targets in aging and degenerative diseases [10, 11].

### Histone post-translational modifications in the aging brain

Post-translational modifications (PTMs) of proteins are pivotal contributors to nervous system aging, regulating chromatin plasticity, gene expression networks, and cellular physiology. Among these, histone PTMs constitute a principal epigenetic regulatory mechanism. Histones (H1, H2A, H2B, H3, H4) form chromatin’s fundamental repeating units through nucleosome assembly and critically regulate transcriptional processes in eukaryotes [12]. Specifically, histone modifications modulate transcriptional repression or activation through alterations in nucleosome stability, chromatin dynamics, and histone-DNA interfaces [12, 13].

The N-terminal tails of histones are hotspots for diverse and site-specific PTMs, including acetylation, methylation, ubiquitination, phosphorylation, and the recently discovered lactylation. These modifications occur on specific amino acid residues [12, 14]. Among these, lysine methylation and acetylation are the most extensively characterized PTMs, with established roles in fundamental processes including transcriptional regulation, DNA repair, cellular differentiation, stress adaptation, aging, and cell division (mitosis/meiosis) [12]. Notably, experimental models, both in vivo and in vitro, report altered levels of histone methylation (e.g., H3K9me3, H4K20me3, H3K27me3, H3K79me3, H3R2me2) and acetylation (e.g., H3K9ac) in the aging brain. These findings suggest that aging instigates changes in histone modifications that propagate aging processes and exacerbate neurodegenerative disease progression [15, 16]. Epigenetic coordination of histone marks involves enzymatic counterregulation, where methylation balance is governed by the interplay between methyltransferases (HMTs) and demethylases (HDMs), and acetylation dynamics by the opposition between histone acetyltransferases (HATs) and histone deacetylases (HDACs) [17], with detailed mechanisms and classifications already reported [18].

Ubiquitination is considered a key mechanism for maintaining protein homeostasis and cell function, including organelle activity and synaptic plasticity of neurons, through the ubiquitin-proteasome system (UPS) and the autophagy-lysosomal pathway [19, 20]. Protein ubiquitination involves a cascade of three active enzymes (E1, ubiquitin-activating enzyme; E2, ubiquitin-conjugating enzyme; and E3, ubiquitin protein ligase), and it is removed by deubiquitinating enzymes (DUBs) [21]. In *C. elegans*, UPS profiling reveals that aging remodels ubiquitination patterns, primarily driven by increased deubiquitinase activity. This shift results in reduced ubiquitination of multiple short-lived and degradable proteins, thereby influencing proteostasis and lifespan regulation [22]. A recent study has shown that the monoubiquitination of histone H2A (H2Aub) is an evolutionarily conserved aging marker that accumulates continuously during the aging process of fruit flies. This phenomenon also exists in other species, including mice and humans. This continuous modification is closely related to enhanced chromatin repression and may promote the aging process by limiting the transcription of genes involved in the oxidative stress response [23]. Furthermore, the decline of UPS activity is regarded as an early event during brain aging, which progressively impairs neuronal proteostasis [24]. Specifically, the abnormal ubiquitination and misfolding of easily aggregating proteins such as tau and α-synuclein (αS) lead to insufficient clearance of these proteins and further result in pathological deposits, causing neuronal aging and death[20]. This represents one of the important mechanisms by which aging contributes to neurodegenerative diseases [25].

Histone phosphorylation regulates the transmission of neuronal signals and monitors the genome in the aging brain, being dynamically regulated by kinases (e.g., ATM/ATR, MSK1, Aurora kinases) and phosphatases to coordinate chromatin accessibility, transcription, and DNA damage responses [26]. During normal aging in humans and mice, phosphorylation of H2AX at Ser139 (γH2AX) progressively accumulates, which reflects an increase in irreparable double-strand DNA break damage and is considered to have a causal relationship with aging [27]. In contrast, H3S10p, a mark associated with activity-dependent transcription and synaptic plasticity, declines in aging mice and is associated with spatial learning and memory deficits in these mice [28]. However, the correlation between this modification and brain aging is less clear, and more research is needed to elucidate the underlying mechanisms in the future.

Further, histone lactylation is a recently discovered PTM, regulated by the levels of lactate in the cell [29]. Histone lactylation and acetylation compete for modification of lysine residues. The enzymes that catalyze (writers) and remove (erasers) lactylation modifications are often the same as histone acetylases and deacetylases [30, 31]. Histone lactylation functions in gene regulation by becoming enriched at the promoter regions of specific target genes across diverse cell types, thereby modulating distinct biological processes [32, 33]. Collectively, the dysregulation of diverse PTMs constitutes a fundamental epigenetic mechanism underpinning brain aging and neurodegeneration, presenting a promising landscape for therapeutic intervention.

## Histone PTM landscapes across aging brain cells

Healthy aging is fundamentally characterized by the accumulation of cellular damage over time, mediated by interrelated mechanisms including metabolic dysregulation, oxidative stress, epigenetic alterations, chronic low-grade inflammation (inflammaging), telomere attrition, and genomic instability [34–39]. This natural process may become harmful when further exacerbated. Epigenetic alterations are widely recognized as a hallmark of aging, with substantial evidence indicating their fundamental contribution to the aging process. First, the accrual of epigenetic alterations during aging drives genomic destabilization and transcriptome dysregulation, constituting core mechanisms of senescence [40]. Second, progressively accumulated epigenetic changes exhibit heritability across generations, thereby influencing aging phenotypes in descendants [41]. Nevertheless, elucidating the causal links between epigenetic drivers and aging remains an active research frontier, with critical implications for developing geroprotective strategies and counteracting associated pathologies. The following sections dissect pathological mechanisms and therapeutic rescue strategies through the prism of histone PTM landscapes.

### Neurons

Age-dependent reduction in histone acetylation remodels chromatin architecture, inducing transcriptional silencing of genomic loci governing neural development, defense mechanisms, plasticity, and memory consolidation. Altered expression of HATs, HDACs, and associated regulatory factors occurs in the aging brain and may drive age-related transcriptional dysregulation [42]. Declining H3K27ac occupancy at gene promoters coincides with age-related suppression of neuronal and synaptic transcripts in the human and murine prefrontal cortex. SAHA-mediated histone deacetylase inhibition ameliorates neuronal degeneration and synaptic dysfunction in aging models, the study further demonstrated that excessive H3K27 acetylation at specific genomic loci suppresses the upregulation of aging-related genes, including key regulators of the NF-κB signaling pathway [43]. Increased HDAC2 binding to neuronal immediate early gene (IEG) promoters during aging reduces H3K9ac and H3K14ac levels, thereby suppressing the expression of these IEGs, which are molecular markers for long-term memory and synaptic plasticity [44].

The age-associated decline in cellular NAD+ levels results in progressive reduction of histone deacetylase Sirtuin 1 (SIRT1) activity. SIRT1 is a regulatory enzyme of the SASP, and its levels and activity decline during aging and cellular senescence, leading to an increase in H4K16ac and H3K9ac, which in turn enhances the activity of the NF-κB transcription factor, which is necessary for the transactivation of SASP [45]. Interestingly, through secreted factors, aged brain cells disseminate senescence to neighboring healthy cells such as endothelial cells, astrocytes, and microglia. Alongside its influence on SASP expression, SIRT1 controls axonal development and synaptic processes, including those governing cognitive function and plasticity, in aging individuals [46–48]. Hippocampal CA1 neurons deficient in SIRT1 show decreased synaptophysin expression and lower dendritic density, indicating that age-related decline in SIRT1 activity may contribute to cognitive impairment in older adults [48]. SIRT1-deficient animals exhibit reduced levels of the transcription factor cAMP response element-binding protein (CREB), compromising its binding to the brain-derived neurotrophic factor (BDNF) promoter, potentially impairing BDNF expression in the brain and ultimately damaging synaptic plasticity [49].

The progressive imbalance in histone acetylation/deacetylation during aging is accompanied by analogous dysregulation of histone methylation. Given methylation’s context-dependent roles in transcriptional activation and silencing, these cumulative changes detrimentally affect cognitive functions and memory processes [50]. H3K4me3 is a histone mark enriched at promoters of neuron-related genes and has been implicated in transcriptional activation [51]. In the CA1 neurons of mice, broad domains of H3K4me3 are associated with increased transcription during memory formation [52]. Compared with newborns, individuals over 60 years of age exhibit a significant reduction in H3K4me3 enrichment at 556 genes in the prefrontal cortex, predominantly associated with neuronal growth, differentiation, and connectivity [53]. At the cellular level, age-related epigenetic changes can be observed, including chromatin rearrangement, which causes heterochromatin to dissociate from the nuclear periphery [54]. Lamin B1 mediates heterochromatin anchoring to the nuclear membrane through preferential binding to the repressive histone modifications H3K9me3 and H3K27me3. Age-related declines in Lamin B1 and the reduction in H3K27me3 and H3K9me3 levels are closely associated with an increase in aging-related and anti-proliferative genes, notably including classic SASP genes [55]. Aging induces overexpression of methyltransferase SUV39H1 in the male mouse hippocampus, concomitant with elevated global and gene-specific methylation. Histone H3K9me3 levels increase at the promoter regions of inducible genes, leading to reduced gene expression and showing a negative correlation with memory performance [56]. Therapeutic targeting of SUV39H1, either as monotherapy or in synergistic combination with other epigenetic regulators, may attenuate pathological memory deficits [57]. Another study found that BAZ-2 (a neuronal epigenetic reader) and EHMT-1 (a histone methyltransferase) drive aging-associated behavioral deterioration in *C. elegans* through mitochondrial dysfunction by downregulation of nuclear-encoded mitochondrial proteins. In murine neurons and human cells, the phylogenetic conservation of this pathway suggests that targeting BAZ-2/EHMT1 may represent a therapeutic strategy for promoting healthy aging [58].

Lactate concentration within the tissue microenvironment serves as a critical diagnostic parameter for microcirculatory efficiency and incipient organ dysfunction, concurrently functioning as a substrate for lactylation modifications [59]. Histone lactylation was first proposed by Zhang in 2019 [29], and substantial research is underway, particularly focusing on the aging brain and Alzheimer’s disease. Lactate plays several crucial roles in neurological health. For instance, intrahippocampal administration of lactate in mice improves spatial memory by upregulating synaptic proteins and inducing protein lactylation [60]. Lactate treatment in the hippocampus of aged mice upregulates angiogenic signaling (via p-AKT/t-AKT, eNOS, and VEGF), mitochondrial markers (including SDHA), and metabolic regulators (including p-CREB/t-CREB, p-HSL/t-HSL, and LDH). Concurrently, it enhances BDNF-associated pathways (involving PGC-1α, SIRT1, and BDNF) and elevates metabolic substrates (namely lactate and pyruvate) [61]. Both studies suggest that increasing lactate levels in hippocampal neurons may improve the aging process by promoting gene expression through histone lactylation. However, an earlier study demonstrated that brain lactate levels (in the cortex and hippocampus) increase during aging, accompanied by an elevated transcriptional ratio of lactate dehydrogenase (LDH) subunits A to B [62], although this study was later shown to potentially contain errors [63]. Therefore, the effects of lactate and lactylation on aging require more in-depth research to elucidate the underlying mechanisms.

### Microglia

Aging is characterized by neuroimmune alterations in the central nervous system, reflecting the coincident progression of microglial dysfunction and cellular aging. With aging, microglia exhibit an impaired capacity to initiate appropriate immune responses and maintain synaptic homeostasis, thereby contributing to age-related cognitive decline and progressive neurodegeneration [64]. Studies have shown that with aging, the levels of acetylation modifications in microglia decrease, disrupting their capacity to respond to oxidative stress and inflammatory responses [65]. For example, aged microglia exhibit significantly reduced expression of acetyltransferases, potentially diminishing intracellular antioxidant enzyme activity and contributing to neuronal damage. Conversely, several deacetylases, notably HDAC1, demonstrate elevated expression in microglia during both in vitro and in vivo aging, correlating with increased biomarkers of microglial senescence [66].

Microglial histone methylation status exhibits age-dependent alterations that can influence glial cell function and phenotype. For example, H3K27me3 is a transcriptionally repressive chromatin modification dynamically regulated by the methyltransferase EZH2 and the demethylases Jmjd3. Tang et al. found that senescent mice exhibit reduced cerebral Jmjd3 expression, concomitant with elevated H3K27me3 levels and an increased ratio of pro-inflammatory M1 to anti-inflammatory M2 microglial markers, and the increase in pro-inflammatory M1 microglia may raise the risk of neuroinflammation and neuronal injury. This suggests that aging is a key factor in the phenotypic transition of microglia [67]. In contrast, other studies have found that in age-related microglial phenotypic changes, aging microglia are not in a state of hyperactivation but instead exhibit reduced ability to mount effective immune responses and become dysregulated [68–70]. Another study on H3K27me3 demonstrates its role in repressing Suppressor of Cytokine Signaling 3 (Socs3) expression. Inhibition of EZH2 reverses this repression, upregulating Socs3 to mediate TRAF6 ubiquitination and subsequent suppression of the Toll-like receptor (TLR)/NF-κB signaling pathway, ultimately attenuating microglial activation and neuroinflammatory responses [71]. Additionally, histone methylation may also influence microglial responses to neural injury, reducing their repair capacity and accelerating neuronal degeneration. Therefore, restoring normal immune function in microglia or promoting the conversion of M1 microglia to M2 microglia to reduce neuroinflammation in the brain could be a viable approach to modulating aging processes and neurodegenerative diseases [72].

### Astrocyte

Astrocytes maintain CNS homeostasis by regulating synaptic glutamate, scavenging free radicals, and producing neurotrophic factors, which are essential for neuronal survival. Conditional astrocyte ablation in adult mice induces severe neurodegeneration [73], highlighting their irreplaceable role. Under inflammatory stimuli, reactive astrocytes secrete pro-inflammatory cytokines (e.g., IL-1β, TNF-α) and increase ROS production, amplifying inflammation and exacerbating neurodegeneration [74]. This reactive astrogliosis is astrocytes’ adaptive response to CNS insults (trauma, infection, misfolded proteins, excitotoxicity), which can also induce astrocyte senescence and accelerate neurodegeneration [4].

The epigenetic regulation of HDAC can coordinate its function by influencing the activation of astrocytes and cytokine production. A study has shown that dimethylfuran treatment reduces HDAC levels in astrocytes and helps inhibit inflammatory responses [75]. LPS-activated microglial conditioned medium led to a reduction in total H3 and H4 acetylation in cultured astrocytes and decreased the expression of the antioxidant factor Nrf2, while the HDAC inhibitor valproic acid alleviated the negative effects of LPS-treated microglial conditioned medium [76]. Drugs such as valproic acid and sodium butyrate have been used to recruit the transcriptional co-activator p300 and upregulate the H3K4me2 modification at the HSP70 promoter, enhancing the expression of neuroprotective HSP70 in rat astrocytes [77].

Collectively, these in vitro findings demonstrate that enhancing histone acetylation in astrocytes can attenuate their pro-inflammatory activity and augment neuroprotective functions. However, there remains a significant gap in our understanding: few studies have investigated the in vivo relevance of histone modifications in astrocytes, including their impact on inflammation- and aging-associated neurodegenerative processes.

### Blood-brain barrier

**Fig. 1:**
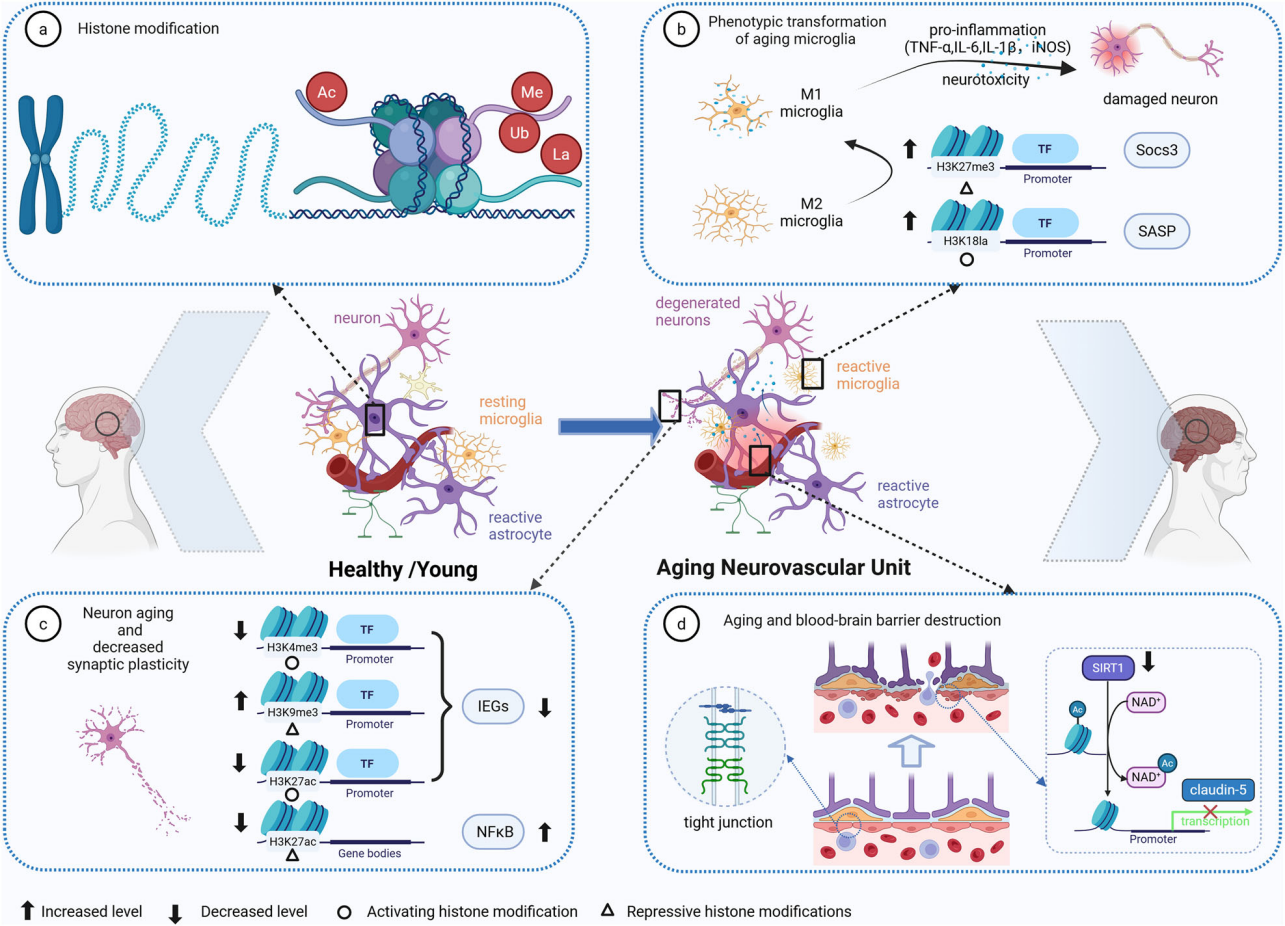
Neurovascular unit dysfunction associated with changes in histone modifications in the context of brain aging. **a** Histone modifications, including acetylation (Ac), methylation (Me), ubiquitination (Ub), and lactylation (La), regulate gene expression and chromatin structure, influencing cellular processes such as aging. **b** During aging, microglial activation induces a phenotypic shift from homeostatic microglia toward M1 and M2 states. M1-polarized microglia release pro-inflammatory cytokines (such as TNF-α, IL-6) that elicit neurotoxicity and neuronal damage. This inflammatory phenotype is associated with alterations in histone modifications (e.g., H3K27me3, H3K18la) at promoters of genes such as *Socs3* and SASP-associated genes, thereby driving neuroinflammatory responses. **c** Neuronal aging is characterized by a decrease in synaptic plasticity, marked by changes in histone modifications such as H3K4me3, H3K9me3, and H3K27ac. These modifications regulate genes related to immediate early genes (IEGs) and NF-κB activation, contributing to neuronal degeneration. **d** Aging also leads to blood-brain barrier (BBB) disruption. A decrease in SIRT1 activity, which is involved in NAD+ metabolism, results in the downregulation of claudin-5, a tight junction protein. This is further influenced by histone acetylation at the *claudin-5* promoter, contributing to the breakdown of BBB integrity during aging.

Neurons, endothelial cells, pericytes, and astrocytes collectively form the blood-brain barrier (BBB), a structurally complex interface that segregates blood from brain parenchyma and precisely controls the neural microenvironment to support neuronal activity. Clinical and experimental data indicate that with aging, BBB integrity gradually declines, potentially leading to cognitive impairments [78]. These impairments are associated with damage to brain endothelial cells and neurovascular unit remodeling, characterized by aging-induced increases in endothelial oxidative stress and a senescence-associated inflammatory microenvironment produced by several types of senescent glial cells. BBB impairment and leakage manifest in the hippocampus, cortex, and corpus callosum, characterized by reduced expression of key tight junction (TJ) proteins (claudin-5, ZO-1, and occludin), a hallmark of BBB aging. BBB disruption further contributes to the initiation and pathogenesis of neurodegenerative disorders; underlying mechanisms have been comprehensively discussed in earlier reviews [79, 80]. Recent studies demonstrate that SIRT1 expression is significantly decreased in brain endothelial cells isolated from aged mice and human tissue. Overexpression of SIRT1 stabilizes claudin-5/ZO-1 interactions, restores claudin-5 levels, and consequently preserves endothelial barrier integrity by attenuating aging-induced hyperpermeability [81]. Extensive research has elucidated the contribution of astrocytic histone methylation and acetylation to neuroinflammation and neurodegeneration, but the contribution of aging astrocytes to BBB damage remains unclear [37]. Currently, research on epigenetic modifications of the BBB has mainly focused on changes after BBB damage in disease states (e.g., stroke, infectious encephalopathy) [82]. However, studies on epigenetic modifications of the BBB in aging are still limited, and detailed research could provide more insights into preventing BBB aging-related damage and disease onset (Fig. 1).

## Histone modification dynamics in age-related neurodegenerative pathologies

Neurodegenerative diseases encompass a heterogeneous group of brain disorders, exemplified by AD, PD, Huntington’s disease (HD), amyotrophic lateral sclerosis (ALS)/frontotemporal dementia (FTD), and multiple sclerosis (MS). Notwithstanding their differences in clinical presentation and underlying pathology, progressive neuronal degeneration leading to structural and functional neurological deficits constitutes a hallmark feature shared across these conditions. Substantial evidence indicates that aging and disruption of histone modifications are critical drivers in the pathogenesis of neurodegenerative diseases. Epigenomic analyses of the aging brain have revealed certain pathways linked to neurodegenerative diseases. Transcriptomic analysis of the human brain demonstrates an age-associated increase in the expression of repressor element 1-silencing transcription factor (REST) targets. REST recruits histone deacetylases, resulting in reduced H3K9ac levels during physiological aging. However, in neurodegenerative diseases such as AD, REST levels decline during aging, resulting in reduced expression of neuroprotective genes including *FOXO* and *BCL2*. This decline further mediates the transcriptional upregulation of genes implicated in AD pathology (such as *PSEN2*) and cell death pathways (including pro-apoptotic factors *BID*, *PUMA*, and *BAX*) [83]. This section summarizes alterations in histone PTMs occurring in common neurodegenerative diseases, such as AD, PD, HD, ALS/FTD, and MS.

### Alzheimer’s disease

**Fig. 2:**
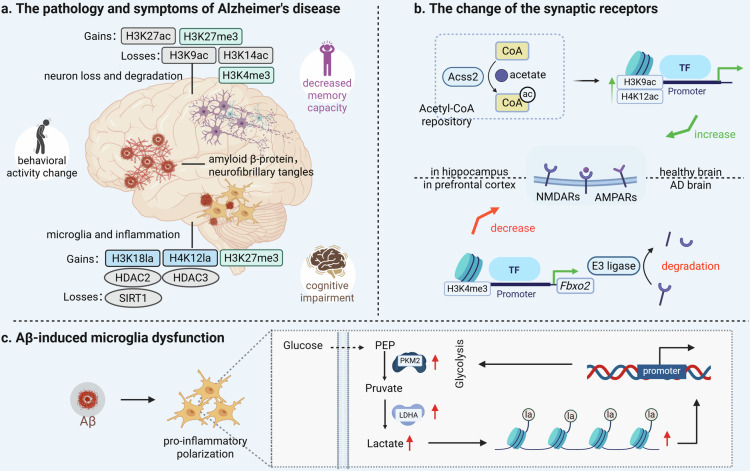
PTM mechanisms associated with Alzheimer’s disease. **a** AD pathology involves dysregulation of histone acetylation and methylation, characterized by specific alterations in neuronal histone modification profiles. These include gains (e.g., H3K27ac, H3K27me3) and losses (e.g., H3K9ac, H3K14ac, H3K4me3), which contribute to neurodegeneration, amyloid-β (Aβ) plaque deposition, and neurofibrillary tangle formation. These changes also contribute to cognitive impairment, behavioral alterations, and microglial activation. **b** Within the healthy brain, synaptic receptors, including NMDARs and AMPARs, are maintained with appropriate histone modifications (e.g., H3K9ac, H4K12ac) and transcriptional activation in the hippocampus and prefrontal cortex. Conversely, the AD brain exhibits significantly reduced NMDAR and AMPAR expression, concomitant with dysregulated acetyl-CoA metabolism, ultimately diminishing synaptic activity. These alterations are mediated by the regulation of histone modifications, which facilitate degradation of key proteins and impair normal synaptic function. **c** Microglial dysfunction induced by Aβ involves alterations in glucose metabolism, highlighted by the upregulation of PKM2 and LDHA in glycolysis and lactate production, which further impacts histone modifications and promoter activities, contributing to AD progression.

AD is a progressive neurodegenerative disorder primarily impacting older adults that is characterized by progressive cognitive decline. Memory impairment represents the defining clinical feature of AD, with disease progression characterized by compromised executive function, language, visuospatial abilities, and olfaction, alongside neuropsychiatric manifestations including affective and behavioral disturbances [84]. The progression of AD is driven by multiple cellular dysfunctions, including mitochondrial dysfunction, dysregulation of gene transcription, protein misprocessing, and synaptic dysfunction [85], with dysregulation of histone modifications being one of the key contributing factors (Fig. 2).

Genomic alterations observed in the hippocampal tissue of patients with AD are associated with abnormal histone modifications, and the resulting mitochondrial dysfunction contributes to the pathogenesis of AD. Studies demonstrate that elevated expression of the neuronal epigenetic regulators BAZ-2B and EHMT1 in the human prefrontal cortex positively correlates with AD progression and negatively correlates with the expression of key mitochondrial proteins. This suggests that these epigenetic changes may exacerbate mitochondrial dysfunction, worsening AD pathology [58] (Table 1).

**Table 1:** Defective histone modifications in neurodegenerative diseases.

Disease	Histone modifications	Model	Gene	Brain region	Histone Modification Level	Effects & References
AD	H3K9me2	5xFAD mice	*GluA2*, *NR2A*, *NR2B*	Prefrontal ectodermal, hippocampus	↑	Abnormal elevation of H3K9me2 leads to down-regulation of glutamate receptor transcription, which in turn causes synaptic dysfunction and cognitive decline. [89]
AD	H3K27ac	Human of AD	*APP*, *PSEN1*, *PSEN2*, *MAPT*	Entorhinal cortex	↑	Differences in H3K27ac modification of genes involved in the progression of Aβ and tau pathology. [101]
AD	H3K27ac	Human of AD	*ABCA7*, *BIN1*, *LRP1B*, *ADAM10*		↑	H3K27ac and CBP/P300 drive a protective transcriptional response in neurons facing amyloidogenic stress, counteracting Aβ accumulation. [100]
AD	Acetylation	App/Ps1 mice		Frontal cortex	↓	Mitochondrial mass reduction and functional impairment, excessive excitation of neuronal networks, and increased neuronal sensitivity to Aβ toxicity. [105]
AD	H3K4me3	P301S tau mice	*Fbxo2*	Prefrontal ectodermal	↑	The elevated expression of Smyd3 in AD leads to an increase in H3K4me3 levels and impaired NMDAR function, causing synaptic dysfunction and cognitive decline. [91]
AD	H4K12la	5XFAD mice	*PKM2*		↑	The glycolysis/H4K12la/PKM2 positive feedback loop exists in AD microglia, which aggravates the metabolic disorder and functional impairment of microglia and promotes the progression of AD. [33]
AD	H4K12la	APP/PS1 mice, BV-2 cells	*NEK7*		↑	Aβ induces an increase in the lactylation level of H4K12, activates the expression of the NEK7 promoter, and thereby promotes pyroptosis of microglia. [109]
AD	H3K18la	APP/PS1 mice, FAD mice, BV-2 cells	*Rela*, *NFκB1*	Hippocampus	↑	Elevated lactate in senescent microglia drives H3K18 lactylation, which activates the NF-κB pathway to upregulate pro-inflammatory SASP components (IL-6/IL-8), thereby accelerating brain aging and AD pathology. [110]
PD	H4K12ac	MP mice/Human of PD	*HVCN1*, *MMP12*, *ATF3*	Dopaminergic neuron cells/substantia nigra	↑	[121]
PD	H3K9ac	N27 dopaminergic neurons, N9 microglia in rat/Human of PD		SNpc (Substantia Nigra pars compacta)	microglia ↑ dopaminergic neurons↓	SIRT2 inhibition demonstrates dual neuroprotective and anti-inflammatory effects. [122]
PD	H3K27me3	Microglial N9 cells in mice/mice	*Arginase1*, *CD206*, *IRF4*		↓	Jmjd3 inhibition drives microglial overactivation, thereby exacerbating dopaminergic neuron loss. [67]
PD	H3K9me2	Primary neurons and mice	*SNAP25*, *PSD95*, *vGLUT1*		↑	The level of H3K9me2 rises, thereby inhibiting the expression of synaptic related genes, causing synaptic damage and motor dysfunction. [125]
PD	H3K4me3, H3K27me3	SH-SY5Y cell, rat	*Fpn1*		↓	GSK-J4 specifically inhibits iron accumulation in dopaminergic neurons through the upregulation of H3K4me3, reduces oxidative stress and provides neuroprotective effects. [134]
HD	H3K9me3	Human of HD, R6/2 mice		Striatum, frontal cortex	↑	Inhibiting Sp1/Sp3-dependent ESET expression suppresses histone H3K9me3, is essential for the survival of neuronal cells. [137]
HD	H3K4me3	Human of HD	*LRRTM2, COX7B*	Frontal cortex	↑	H3K4me3 loses the function of transcriptional activation at the proximal promoter, while the aberrant activation of the distal peak antagonates the PRC2 silencing complex, resulting in a bidirectional imbalance of epigenetic regulation. [139]
HD	H3K4me3, H3K27ac	Human of HD	*NEUROD1*, *GAD1*	Mixed nerve cells	↓	Mutant huntingtin disrupts neurodevelopmental pathways and epigenetic regulation, causing a maturation deficit in HD neural cells. [141]
ALS/FTD	H3K9me3, H3K27me3, H3K79me3, H4K20me3	*C9orf72* repeat expansion carriers	*C9orf72*	Prefrontal cortex and cerebellum	↑	Increased repressive methylation marks mediate the downregulation of *C9orf72* expression. [158]
ALS/FTD	H3K27ac	Human of ALS		Motor cortex and frontal cortex	↑	H3K27ac is significantly increased in astrocytes and is linked to astrocyte activation. [161]
ALS/FTD	H3K36me3	Human of ALS/FTD		Cerebral cortex	↓	The loss of H3K36me3 correlates with impaired RNA polymerase II processivity and pervasive splicing defects. [163]
ALS/FTD	H4R3me2	FUS transgenic mice		Spinal motor neuron	↓	Redistribution of FUS to the cytoplasm leads to depletion of PRMT1 in the nucleus. Hypomethylation of H4R3 results in decreased levels of H3K9ac and H3K14ac, thereby suppressing transcription. [166]
MS	Acetylation	Human of MS	*TCF7L2*	Frontal lobe white matter	↑	The levels of transcriptional repressors for oligodendrocyte differentiation (such as TCF7L2, ID2 and SOX2) are elevated, and they are positively correlated with the duration of MS. [183]

HDAC2 upregulation and Tip60 reduction, along with transcriptional dysregulation-mediated epigenetic disruption of neuronal genes, represent the initial causal events in the AD genome-wide landscape [86]. Research by M. K. Thakur has established that hippocampal upregulation of histone deacetylase HDAC2 in aged mice is positively associated with impaired recognition memory [87]. Further studies show that HDAC2 reduces the levels of H3K9ac and H3K14ac at the promoters of IEGs in aged mouse neurons. Furthermore, suppressing HDAC2 can restore epigenetic modifications, IEG expression, and memory in aged mice [44]. A portion of the nuclear acetyl-CoA pool is locally generated by acetyl-CoA synthetase 2 (ACSS2), and ACSS2 expression is downregulated in the brains of FAD model mice and AD patients. This leads to a reduction in histone acetylation (H3K9ac and H4K12ac) at the promoters of ionotropic glutamate receptor genes, further aggravating synaptic plasticity impairment in AD mice [88].

The accumulation of misfolded proteins during aging represents a hallmark feature of AD, notably characterized by hyperphosphorylated tau contributing to neurofibrillary tangles and Aβ plaque deposition. This not only causes synaptic dysfunction but also leads to dysregulation of histone modifications. In tauopathy mouse models, euchromatin histone methyltransferase 2 (EHMT2) expression is notably elevated in the prefrontal cortex (PFC), resulting in increased deposition of the repressive histone mark H3K9me2 [89]. This suppresses the expression and function of AMPA and NMDA receptors, contributing to cognitive deficits in aged FAD mice. Treatment with the EHMT inhibitor UNC0642 can reverse this memory deficit [90]. Histone H3K4me3 levels are significantly increased in the PFC of tau mouse models, which activates the transcription of the downstream ubiquitination-related gene *Fbxo2*, leading to increased degradation of NMDA receptors and damaged synaptic function [91]. Acute inhibition of histone methyltransferase Smyd3 with BCI-121 ameliorates cognitive deficits and restores both the function and expression of synaptic NMDARs in pyramidal neurons [91].

A genome-wide epigenomic association study in the PFC of aged humans indicated that tau protein load affects 5,990 of the 26,384 H3K9ac domains in the epigenome, altering chromatin structure. This change can be reversed with a heat shock protein 90 (Hsp90) inhibitor, but the deeper impacts of chromatin changes require further investigation [92]. It is noteworthy that histone modifications are not the only mechanism of epigenetic regulation in neurodegenerative diseases. Post-translational modifications of pathological proteins (such as tau and Aβ) themselves also play critical roles in disease progression and exhibit profound crosstalk with histone modification networks. Targeting these non-histone modifications, particularly abnormal acetylation, phosphorylation, and other changes in pathological proteins, represents a highly promising therapeutic strategy.

As research on tau PTMs increases, acetylated tau (which promotes tau aggregation and inhibits its binding to microtubules) has been identified as a major PTM that critically influences both physiological tau function and pathological processes [93]. HDAC6 modulates tau pathology not only by deacetylating tau but also by suppressing its hyperphosphorylation within the microtubule-binding domain. Consequently, HDAC6 deficiency exacerbates Alzheimer’s disease-like neuropathology in PS19 mice [94]. H. Choi demonstrated that acetylated tau recruits chaperone proteins (including Hsp40, Hsp70, and Hsp110), forming a complex with newly implicated tau E3 ligases such as UBE2O and RNF14 to facilitate proteasomal degradation of pathological tau [95]. However, Benjamin et al. demonstrated that acetylated tau is selectively targeted for degradation through both macroautophagy and endosomal microautophagy, and that inhibiting chaperone-mediated autophagic degradation enhances the intercellular propagation of pathogenic tau in mouse models, accelerating disease progression [96]. While these findings are still debated, therapeutic strategies targeting tau post-translational modifications show considerable development potential. Notably, the development of novel anti-acetylated tau monoclonal antibodies is underway [93]. HDAC6-dependent monitoring mechanisms inhibit the accumulation of abnormally modified tau, thereby preventing the progression of AD and related tauopathies [97]. Additionally, in microglial studies, reducing lactylation at the tau K677 site in mouse microglia can lower ferritin autophagy and ferroptosis by modulating the MAPK signaling pathway, leading to improved memory and learning and reduced neuronal damage [98].

Similarly, changes in PTMs also promote the deposition of Aβ. Multi-omics analysis revealed significantly increased levels of CBP/p300 and TRRAP in the brains of AD patients compared to healthy aging controls. This elevation promotes global accumulation of H3K27ac and H3K9ac, disrupts transcriptional regulation and chromatin feedback loops, and activates disease-promoting pathways, including inflammation and cell cycle reactivation. Notably, experimental elevation of H3K27ac or H3K9ac in animal models exacerbates Aβ-induced neurodegeneration [99]. On the other hand, H3K27ac at the promoter of Aβ clearance genes in the brains of elderly AD patients is significantly elevated. In vitro studies have shown that inhibition of the acetyltransferase CBP/p300 reduces H3K27ac and promotes Aβ deposition, suggesting the presence of compensatory genetic programs responding to AD-related damage [100, 101]. Therefore, the same epigenetic modifications concurrently regulate multiple genes that exert divergent effects on disease pathogenesis, necessitating further investigation to identify precise therapeutic targets. Regarding Aβ, Li’s study indicates that the deacetylase SIRT2 accumulates in the aging brain. SIRT2 mediates the deacetylation of K132 and K134 on APP, promoting Aβ formation [102]. In HT22 mouse hippocampal neuronal cells, Aβ inhibits the deacetylase SIRT1 and promotes the expression of SIRT2. Overexpression of SIRT1 can inhibit the deacetylation of APP by SIRT2, reducing Aβ and preventing neurotoxicity [103]. Li further demonstrated that SIRT6 deacetylates specific lysine residues (K649, K650, K651) on APP, promoting its ubiquitination and thereby facilitating proteasomal degradation [104]. Another study showed that Aβ inhibits the expression of the deacetylase SIRT3, increasing mitochondrial protein acetylation in neuronal cells during aging, impairing mitochondrial function, and accelerating the pathological progression of Aβ [105].

Datta reported that HDAC1/HDAC2 knockout promoted microglial phagocytosis of Aβ and rescued cognitive deficits in an AD mouse model [106]. In a related finding, microglial activation was found to mediate HDAC2 overexpression, leading to reduced histone acetylation, transcriptional repression of BDNF and c-fos, and consequent memory impairment [107]. Furthermore, HDAC3 overexpression in APP/PS1 mice increases Aβ burden, activates microglia, and diminishes hippocampal dendritic spine density, whereas lentivirus-mediated HDAC3 inhibition effectively attenuates microglial activation, enhances cognitive function, and ameliorates key AD-related neurodegenerative processes. These findings suggest that inhibiting HDAC1, HDAC2, and HDAC3 can reduce microglial activation, promote Aβ clearance, and potentially reverse the pathogenesis of AD [108].

In microglia proximal to Aβ plaques, elevated levels of lactate-dependent histone lactylation H4K12la are observed in both AD mouse models and human postmortem brain samples. This modification is enriched at glycolytic gene promoters, facilitating transcriptional activation and enhanced glycolytic flux. This H4K12la/glycolysis/PKM2 positive feedback loop thereby perpetuates metabolic dysregulation and pro-inflammatory polarization in microglia during AD pathogenesis. This study indicates that metabolic disorders play a significant role in the neuroinflammation and early stages of AD, pointing to a new direction for the early intervention of AD [33]. In another study, H4K12la was shown to upregulate the expression of NEK7, a serine/threonine kinase. This cascade subsequently activates the NLRP3 inflammasome, thereby triggering neuroinflammation in mice, which leads to neural tissue damage, functional impairment, and deficits in spatial learning and memory [109]. Elevated lactate levels were similarly observed in microglia and hippocampal tissues from both physiologically aged mice and AD models, resulting in increased pan-histone lysine lactylation (notably H3K18la and Pan-Kla). This histone lactylation directly activates NF-κB signaling, inducing expression of SASP components such as IL-6 and IL-8, thereby contributing to accelerated brain aging processes and AD pathology [110]. This suggests that inhibiting histone lactylation modifications may have potential therapeutic benefits. On the other hand, exercise or sodium lactate injection, which increases lactylation of histone H3 in microglia, has been shown to improve hyperactivation, promote an anti-inflammatory/repair phenotype, reduce neuroinflammation, and enhance cognitive function [111], suggesting the multiple effects of histone lactylation at different modification sites. Therefore, further research into the effects of lactate and lactylation modifications on the central nervous system is needed.

In AD, alterations in histone modifications associated with phosphorylation and ubiquitination have also been reported. In the 5XFAD mouse model, which produces high levels of Aβ, levels of H3S57p and H3T58p are significantly reduced. The loss of phosphorylation at these residues promotes heterochromatin formation and leads to decreased gene expression [112]. Ming et al. reported increased phosphorylation of histone H2A.X in astrocytes from AD brains, indicating the presence of DNA damage associated with astrocytic dysfunction [113]. Furthermore, mass spectrometry–based analyses of frontal cortex tissue from human AD donors revealed increased levels of ubiquitinated H2BK120 in AD, although the functional significance of this modification requires further investigation [114].

### Parkinson’s disease

PD is characterized by degeneration of substantia nigra neurons, resulting in striatal dopamine depletion and formation of Lewy bodies, which are a type of intracellular protein aggregate [115]. PD represents a heterogeneous and complex disorder arising from multiple genetic, epigenetic, and environmental factors. It manifests through both motor dysfunction, including bradykinesia, rigidity, postural instability, and tremor, and non-motor manifestations, such as sleep disturbances, olfactory deficits, oculomotor abnormalities, neuropsychiatric features, and cognitive decline [116]. The pathophysiology of PD encompasses multiple interconnected mechanisms, notably the aggregation of α-synuclein oligomers, dysfunction in mitochondrial autophagy, exacerbated oxidative stress, dysregulation of calcium homeostasis, impairment of axonal transport, and augmented neuroinflammation [117]. Existing treatments for PD provide only symptomatic relief and a modest slowing of progression; consequently, the pursuit of curative or neurorestorative interventions constitutes a fundamental research priority.

Epigenetic dysregulation, which drives alterations in gene expression, represents a key pathophysiological factor in PD. PD patients show epigenetic changes and transcriptional upregulation of the DNA hydroxylase TET2, leading to cell cycle arrest and cell death. Studies suggest that overexpression of the deacetylase SIRT1 can induce the deacetylation of TET2 and promote its degradation, thereby reversing cell death [118]. SIRT1 can also upregulate PGC-1α expression and promote mitochondrial biogenesis; knocking out SIRT1 further exacerbates cellular damage. The age-related decline in SIRT3-mediated neuroprotection exacerbates mitochondrial oxidative stress in the PD brain, contributing to dopaminergic neurodegeneration in the substantia nigra pars compacta (SNpc) [119]. Another genome-wide analysis of PD brains revealed elevated levels of H3K27ac, particularly at genes associated with the disease (including *SNCA, PARK7, PRKN*, and *MAPT*) [120].

Degeneration of dopaminergic (DA) neurons is also closely linked to histone PTMs. M. Huang reported elevated H4K12ac deposition in a cellular PD model following mitochondrial stress, mirroring the significant increase detected in postmortem PD patient brains. RNA-seq results further confirmed the association of energy metabolism, mitochondria, and dopamine with the PD model in neurodegeneration [121]. Y. Tang et al. demonstrated that aged mouse brains exhibit reduced levels of the demethylase Jmjd3, concomitant with elevated H3K27me3 and an increased M1/M2 microglial polarization ratio, a phenomenon associated with augmented neuronal death in the PD model. They further demonstrated that reducing H3K27me3 enhanced the polarization of M2 microglia, which reduced dopaminergic neuronal death [67].

In vitro experiments by Harrison et al. demonstrated that degenerating dopaminergic neurons exhibited reduced histone acetylation, whereas activated microglia showed elevated histone acetylation. These epigenetic alterations were associated with dopaminergic neuron degeneration, microglial infiltration, and microglial activation. This study also confirmed that the histone deacetylase SIRT2 plays a key role in this process [122]. Similar findings were observed in astrocyte research, where the use of three HDAC inhibitors increased histone H3 acetylation at the gene promoters of glial cell-derived neurotrophic factor (GDNF) and BDNF in astrocytes and enhanced the expression of GDNF and BDNF, protecting DA neurons in midbrain neuron-glial cell co-cultures [123]. In both PD mouse models and postmortem human PD brain tissue, p300-mediated H3K27ac deposition was observed, which augmented microglial responses to immunogenic stimuli and culminated in secondary neural damage. GNE-049 inhibited H3K27ac deposition, which was shown to prevent the formation of primary immune memory and reduce the subsequent secondary inflammatory response, thereby slowing PD progression [124].

αS is a critical mediator of histone modification dysregulation in PD. One study showed that pre-formed fibrils (PFF) of α-synuclein increased the expression of histone methyltransferases EHMT1 and EHMT2, which led to elevated levels of H3K9me2 at the gene promoters of synaptic-related proteins, resulting in neuronal synapse loss and reduced expression of synaptic proteins, ultimately causing motor deficits. Treatment with the EHMT1/2 inhibitor A-366 suppressed synaptic damage and motor dysfunction in PFF-injected mice [125]. Therefore, reducing αS deposition is a critical approach for treating PD-related histone dysregulation. αS interacts with H3 and inhibits histone acetylation. T. F. Outeiro’s research showed that the deacetylase SIRT2 deacetylates αS at K6 and K10, which promotes its aggregation in the cytoplasm and exacerbates its cytotoxicity [126]. SIRT2 inhibition also attenuated αS-induced toxicity and modified inclusion body morphology in a cellular model of PD [127].

In addition, emerging evidence indicates that tau aggregation and deposition also contribute to the pathogenesis of PD [128]. Unlike the widespread distribution of tau pathology observed throughout the brain in AD, tau pathology in PD and Parkinson’s disease dementia (PDD) is largely restricted to dopaminergic neurons within the nigrostriatal system [129]. Notably, tau has been found to colocalize with αS in PD brains, and their interaction synergistically disrupts cytoskeletal integrity, leading to PD-associated neuronal death [130, 131]. One study demonstrated interactions between tau and α-tubulin, as well as between αS and α-tubulin, in cellular models of PD, providing evidence that both tau and αS function as microtubule-associated proteins. Inhibition of SIRT2 was functionally linked to increased α-tubulin acetylation and improved microtubule dynamics, accompanied by reduced tau phosphorylation and increased αS acetylation, as well as enhanced binding of tau and αS to microtubules [132]. Finally, although widespread dysregulation of histone post-translational modifications has been reported in PD, histone modifications specifically associated with tau pathology remain insufficiently characterized and warrant further investigation.

During aging, iron accumulation in the substantia nigra leads to neuronal ferroptosis and is considered a significant hallmark of PD [133]. Therefore, shifting accumulated intracellular iron to the extracellular space has become a research focus. In vivo studies demonstrated that the histone demethylase inhibitor GSK-J4 crosses the BBB, targets dopaminergic SH-SY5Y neuronal cells, upregulates H3K4me3, and subsequently enhances ferroportin-1 expression, thereby reducing intracellular iron accumulation, mitigating oxidative damage, and conferring neuroprotection [134]. Suv39h1-mediated H3K9me3 represses *Tfr1* transcription to limit neuronal iron uptake, thereby acting as a protective mechanism against neuronal ferroptosis [135].

### Huntington’s disease

HD, an autosomal dominant neurodegenerative disorder manifesting as progressive cognitive and motor decline, arises from a CAG trinucleotide repeat expansion in the *HTT* gene, which produces a mutant huntingtin protein (mHtt) responsible for the neurological dysfunction [136]. The chromatin changes observed in HD may be induced by these expansions. In both HD models and human HD brain tissues, an increase in H3K9me3 heterochromatin domains has been noted. Combined micafungin and cystamine treatment ameliorates histone H3K9 hyper-trimethylation and attenuates behavioral and neuropathological deficits in a HD mouse model [137], and chromatin remodeling drugs like nogalamycin show similar therapeutic effects [138].In another study, analysis of post-mortem human prefrontal cortex from HD patients and controls revealed a neuron-specific reduction of H3K4me3 (which is associated with gene activation), with many of the altered H3K4me3 peaks located near genes involved in synaptic function [139]. The histone acetyltransferase CBP is mislocalized to mutant proteins in HD cultured cells, mouse models, and post-mortem HD brains [140]. Other studies reported changes in HDAC1, HDAC5, and HDAC6 in the brains of HD patients, leading to reduced acetylation of H3K9 and H3K27, further contributing to neuronal dysfunction [141]. Although various HDAC inhibitors demonstrate therapeutic efficacy in ameliorating HD symptoms in animal models [18], recent studies reveal discordant histone acetylation patterns between these models and human post-mortem HD brains. The authors suggest that treatments for HD patients may require alternative epigenetic targets [142]. The relationship between HD and aging is also a worthwhile area of exploration. In cases of HD with age-of-onset variations that cannot be explained by the Huntington gene alone, genetic or environmental factors may be contributing factors. Aging-related increased risk of onset should not be overlooked as an important factor [143]. On the other hand, the epigenetic age (DNA methylation) of HD patient brain tissue is greater than the patients’ chronological age, suggesting an accelerated aging effect [144], although the detailed mechanisms in the realm of histone modifications remain unclear.

Recent studies have revealed the presence of aggregated tau inclusions in the brains of patients with HD. These tau alterations include increased total tau levels, the emergence of truncated tau species, and tau hyperphosphorylation [145]. Blum and colleagues proposed that mHtt induces tau hyperphosphorylation, subcellular redistribution, and aggregation through direct protein–protein interactions [146]. In addition, inactivation of tau phosphatases appears to be a more likely contributor to abnormal tau phosphorylation, as downregulation of phosphatases such as PP1, PP2A, and PP2B has been detected across multiple HD mouse models [146, 147].

It is worth noting that tau protein pathology is a common pathological marker in AD, PD and HD. Although multiple studies have independently reported the presence of histone acetylation defects and changes in inhibitory markers in AD, PD, and HD, there is still limited evidence directly comparing the epigenomic regulatory mechanisms of tau protein pathology in these diseases. Currently, there is a lack of systematic cross-disease analysis, a gap merits further investigation.

### Amyotrophic lateral sclerosis/frontotemporal dementia

ALS and FTD are clinically distinct neurodegenerative disorders. ALS is clinically defined by the progressive degeneration of upper and lower motor neurons, leading to muscle weakness and paralysis [148], whereas FTD represents a heterogeneous group of syndromes primarily characterized by behavioral, executive, and language impairments resulting from degeneration of the frontal and temporal cortices [149]. Despite their distinct clinical presentations, partial overlap exists: ALS patients may exhibit cognitive impairment, while a subset of FTD patients develops motor symptoms [150]. From a neuropathological perspective, both disorders are characterized by cytoplasmic inclusions composed of aggregation-prone proteins, most commonly TDP-43 or FUS, accompanied by nuclear depletion, neuronal loss, gliosis, and region-specific brain atrophy [151]. Consequently, ALS and FTD are increasingly recognized as a unified neurodegenerative disease spectrum with overlapping clinical, genetic, and molecular features [152]. Shared pathogenic mechanisms underlying the ALS/FTD spectrum include defects in RNA metabolism, impairment of proteostasis, mitochondrial dysfunction, compromised DNA damage repair, and chronic neuroinflammation [148, 149]. Most ALS/FTD cases are sporadic, whereas familial ALS/FTD is associated with mutations in multiple genes. *TARDBP*, *FUS*, and *C9orf72* (C9) are among the key genes implicated in ALS/FTD [153], and mutations in these genes promote protein misfolding and aggregation. Age is a major risk factor for ALS/FTD, as the incidence of both diseases increases with advancing age, particularly after 60 years, and the age of onset is associated with disease severity and progression [154]. Currently, no curative therapies are available for ALS or FTD. Treatment strategies primarily focus on symptom management: riluzole and edaravone have been approved for ALS, whereas therapeutic approaches for FTD remain limited to the management of behavioral symptoms and cognitive decline [149, 155]. Emerging evidence links epigenetic dysregulation, particularly aberrant histone post-translational modifications, to ALS/FTD-related pathology, offering potential avenues for therapeutic intervention [156].

A GGGGCC hexanucleotide repeat expansion within the first intron of C9 represents one of the most common genetic causes of ALS and FTD. The pathogenic mechanisms are thought to involve a combination of loss of normal C9 function and toxic gain-of-function effects, including repeat RNA–mediated sequestration of RNA-binding proteins and the production of aggregation-prone dipeptide repeat proteins through repeat-associated non-ATG translation [157]. Alterations in histone methylation constitute a core epigenetic feature of C9-associated FTD/ALS and other pathogenic contexts. In postmortem human frontal cortex and cerebellum from C9 repeat expansion carriers, increased levels of repressive trimethylation marks, including H3K9me3, H3K27me3, H3K79me3, and H4K20me3, have been observed and correlate with reduced C9 mRNA expression. Treatment with a demethylating agent (5-aza-2′-deoxycytidine) reduces these trimethylation marks and partially restores C9 expression in patient-derived fibroblasts [158]. In a C9 bacterial artificial chromosome (BAC) transgenic mouse model, enrichment of H3K9me3 at the human transgene promoter is associated with transcriptional repression and age-dependent locus silencing, consistent with epigenetic signatures observed in patients and suggesting early epigenetic programming driven by repeat toxicity [159]. Paradoxically, in the same C9 BAC model, global levels of heterochromatic H3K9me3 are reduced in astrocytes and neurons of the spinal cord, motor cortex, and hippocampus, correlating with neuronal loss and cognitive impairment. These findings indicate a redistribution of repressive methylation during neurodegeneration rather than uniform hypermethylation [160]. Single-cell epigenomic analyses of brains from C9-associated ALS and FTD patients further reveal increased H3K27ac at promoter and enhancer regions in glial cells, which is tightly linked to transcriptional activation of immune- and inflammation-related genes. This epigenetic remodeling is comparatively limited in neurons, highlighting glia-specific chromatin activation as a shared pathogenic epigenetic mechanism in ALS/FTD that may drive chronic neuroinflammation and disease progression [161]. Additionally, studies in C9 patient-derived neurons demonstrate that repeat RNA and dipeptide repeat proteins indirectly disrupt chromatin regulatory complexes, such as DAXX/ATRX, leading to hypoacetylation and hypermethylation at the *C9orf72* promoter and across the genome, and consequent chromatin instability [162].

TDP-43, encoded by *TARDBP*, is a nuclear RNA/DNA-binding protein that regulates transcription, pre-mRNA splicing, and RNA transport. In ALS/FTD, TDP-43 undergoes nuclear depletion and cytoplasmic aggregation, leading to widespread RNA dysregulation and neuronal toxicity [151]. In TDP-43 proteinopathies, alterations in PTMs arise primarily through disruption of nuclear RNA–chromatin coupling and transcriptional regulation. Studies using yeast, neuronal cell lines, transgenic mice expressing wild-type or mutant TDP-43, as well as postmortem cortical tissue from ALS/FTD patients, consistently report transcription elongation–associated changes in histone methylation, most notably a reduction in H3K36me3. This loss correlates with impaired RNA polymerase II processivity and pervasive splicing defects [163]. Concurrently, histone acetylation patterns are dysregulated, with yeast and mammalian models exhibiting increased H4K12ac and H4K16ac, indicating compensatory or maladaptive chromatin opening in response to TDP-43–induced transcriptional stress [163]. Furthermore, in mouse models of FTD, TDP-43–mediated pathology is associated with reduced activity of HDAC1, a class I histone deacetylase [164]. Collectively, TDP-43 proteinopathy induces widespread alterations in histone PTMs and chromatin-modifying factors, reflecting genomic instability and progressive heterochromatin disorganization.

Fused in sarcoma (FUS), encoded by *FUS*, is a multifunctional RNA-binding protein involved in transcriptional regulation, DNA damage repair, and RNA metabolism. ALS/FTD-associated mutations promote its cytoplasmic mislocalization and aggregation, resulting in toxic gain-of-function effects and loss of nuclear homeostasis [165]. In yeast models overexpressing FUS, decreased levels of H3K14ac, H3K56ac, H4R3me2, and H3S10ph, histone marks associated with transcriptional activation, have been reported [163]. Similar alterations have been observed in animal models. In motor neurons of FUS transgenic mice, cytoplasmic mislocalization of FUS leads to nuclear depletion of protein arginine methyltransferase 1 (PRMT1), which catalyzes H4R3me2. Loss of this histone mark is associated with reduced transcriptional activity [166]. Moreover, in the spinal cord and cortex of FUS transgenic mice, FUS pathology is closely linked to global hypoacetylation of histone H3, with reduced acetylation correlating with repression of metabolic and neuronal gene programs. Notably, treatment with the HDAC inhibitor ACY-738 restores histone acetylation and significantly improves motor phenotypes and survival [167]. Additional studies indicate that acetylation of FUS itself regulates its nuclear localization and aggregation propensity, suggesting that post-translational modification of FUS directly contributes to the pathophysiology of FUS-mediated ALS/FTD [168].

In summary, across the ALS–FTD spectrum, genetic mutations and proteinopathies converge on dysregulation of histone post-translational modifications, leading to persistent transcriptional dysfunction. These findings position histone-modifying enzymes and chromatin states as mechanistically grounded and potentially reversible therapeutic targets for ALS/FTD.

### Multiple sclerosis

MS is a chronic immune-mediated disease of the central nervous system that typically affects young adults and manifests as relapsing or progressive neurological symptoms, including sensory loss, motor dysfunction, visual impairment, and cognitive decline. The prevalence of MS is higher in women and in populations residing at higher latitudes. Clinically, MS presents with several disease courses: relapsing–remitting MS (RRMS, the most common initial form), secondary progressive MS (SPMS), and primary progressive MS (PPMS) [169]. The exact etiology of MS remains unclear; however, it is widely accepted that the disease arises from an interplay between genetic susceptibility, most notably specific *HLA-DRB1* variants, and environmental factors such as Epstein–Barr virus infection, vitamin D deficiency, smoking, and obesity. Together, these factors trigger aberrant immune responses in genetically predisposed individuals [170]. Pathologically, MS is characterized by multifocal inflammatory demyelinating lesions accompanied by axonal injury, oligodendrocyte loss, and widespread gray matter neurodegeneration. These processes are driven by immune cell infiltration, disruption of the blood–brain barrier, mitochondrial dysfunction, and chronic microglial activation [171]. Current therapeutic strategies primarily focus on immunomodulation or immunosuppression to reduce relapse rates and inflammatory activity, including interferon-β, B cell–targeting monoclonal antibodies (e.g., anti-CD20), and sphingosine-1-phosphate receptor modulators. However, these treatments show limited efficacy in preventing long-term neurodegeneration and progressive disability [170]. Increasing evidence indicates that alterations in PTMs contribute to MS pathogenesis by shaping immune responses and modulating the functional states of central nervous system cells [172].

A hallmark feature of MS and experimental autoimmune encephalomyelitis (EAE) is immune-mediated, inflammation–driven myelin damage and axonal injury that begin at disease onset. Autoreactive myelin-specific CD4⁺ T cells, their differentiated T helper (Th) subsets (Th1, Th2, Th17, and regulatory T cells [Tregs]), as well as monocytes/macrophages, are considered key contributors to MS pathogenesis [173, 174]. Activation of autoreactive CD4⁺ T cells and their differentiation toward pro-inflammatory Th1 or Th17 phenotypes represent critical early events in MS, whereas Th2 polarization and Treg expansion are associated with reduced inflammation and clinical improvement in MS patients [173]. In addition, the balance between classically activated (M1) and alternatively activated (M2) monocytes/macrophages further shapes immune responses during disease progression: M1-polarized monocytes/macrophages promote Th1-skewed immunity, whereas M2-polarized cells favor Th2-type immune responses [175]. Studies have shown that histone deacetylase (HDAC) inhibitors suppress Th1-derived pro-inflammatory cytokine production, such as interleukin-2 (IL-2), and promote the differentiation of Th1 cells toward a Treg phenotype through upregulation of FOXP3 [176]. Consistently, in HDAC11-deficient models, loss of HDAC11 restricts monocyte and dendritic cell infiltration into the central nervous system, thereby attenuating demyelination and reducing disease severity [177]. Moreover, multiple studies have demonstrated that HDAC inhibition contributes to the restoration of immune homeostasis in EAE models [178, 179]. In parallel, histone methylation also plays a critical role in shaping the inflammatory milieu in MS. Mice deficient in the histone demethylase Jmjd3 exhibit resistance to EAE induction, and the Jmjd3-specific inhibitor GSK-J4 markedly suppresses Th17 cell differentiation in vitro [180].

Another major feature of MS is impaired remyelination, which results from defective differentiation of endogenous oligodendrocyte progenitor cells (OPCs). In normal, undifferentiated OPCs, histone acetylation levels are relatively high, a state that favors the expression of transcriptional repressors of myelin genes [181]. Conversely, removal of acetylation marks by histone deacetylases (HDACs) promotes OPC differentiation and remyelination, and remyelination failure in the aged demyelinated brain is associated with reduced efficiency of HDAC recruitment. Consistent with this, pharmacological inhibition of HDACs prevents the differentiation of progenitor cells into mature oligodendrocytes [182]. Notably, although a large number of oligodendrocytes with histone deacetylation are observed in early MS lesions, oligodendroglial lineage cells in the normal-appearing white matter (NAWM) of patients with chronic MS exhibit significantly elevated levels of histone acetylation [183]. Taken together, these findings indicate that while HDAC-mediated oligodendrocyte differentiation may exert beneficial effects in MS by facilitating remyelination, HDAC-dependent T cell activation may initiate pathogenic immune responses and exacerbate disease severity.

## The comparison of aging brains and diseases among humans

As the research on PTMs in the aging human brain continues to expand, we compare PTMs in the aging human brain and in diseases, with a focus on AD, to delineate their similarities and distinctions in this section.

A ChIP-seq study of the human temporal cortex demonstrated that H4K16ac levels were broadly increased during normal aging. These gains were enriched at genes involved in neuronal maintenance and stress responses, suggesting a chromatin-based adaptive response in healthy aging [184]. In contrast, AD brains exhibited widespread loss of H4K16ac at many of the same genomic loci [184].

Systematic ChIP-seq analyses of the human cortex revealed that aging is associated with the loss of broad gene-body H3K27ac domains, particularly at inflammation-related genes. This reduction correlated with increased expression of inflammatory genes, consistent with age-associated inflammaging. The same study further reported decreased H3K27ac levels at promoters of neural function–related genes, accompanied by reduced expression of the corresponding transcripts [43]. In contrast, integrated epigenomic analyses of AD postmortem brains identified context-dependent gains of H3K27ac at disease-associated promoters and enhancers, including loci involved in chromatin regulation and immune signaling [99].

A study of the human prefrontal cortex demonstrated an age-associated decline in H3K9ac at promoters of neuronal and synaptic genes, which correlated with reduced transcription of plasticity-related genes [185]. In AD, integrated epigenomic analyses revealed increased H3K9ac levels at specific AD-associated genomic regions implicated in disease-related pathways and transcriptional dysregulation [99].

Recent studies identified increased H3K18la in the aged human cortex, linking lactate accumulation to activation of inflammatory gene programs and suggesting a metabolic–epigenetic mechanism underlying inflammaging. Elevated H3K18la was likewise observed in microglial cells from AD brains, where it was associated with upregulation of genes related to inflammatory aging [110].

As noted above, H3K4me3 enrichment at genes involved in neuronal development was reduced in the aged human brain, whereas increased H3K4me3 was observed at other loci associated with transcriptional regulation. These findings indicate substantial remodeling of the H3K4me3 landscape during brain aging, the functional consequences of which appear to be context-dependent [53]. In AD, overall H3K4me3 levels were decreased, particularly at promoter regions of genes implicated in AD-related pathways, including glutamatergic receptor signaling [186].

Overall, accumulating evidence suggests that aging is accompanied by substantial remodeling of histone post-translational modifications in the human brain, which may reflect adaptive chromatin responses to physiological stress. In contrast, AD exhibits more pronounced and dysregulated PTM alterations at disease-relevant loci, potentially contributing to pathological transcriptional changes. However, the number of systematic studies directly comparing age-associated and disease-related PTM changes in human brain tissue remains limited, and available datasets are often restricted by sample size, brain region specificity, and methodological heterogeneity. Consequently, this review is constrained by the scope and variability of current evidence, underscoring the need for more integrative, large-scale epigenomic analyses to clarify causal relationships and determine regional specificity.

## Therapeutic prospects and challenges

### Pharmacological targeting of histone-modification

The reversibility of histone modifications in diseases provides a theoretical basis for clinical treatment. As mentioned above, HDACs are among the most influential epigenetic mechanisms in brain pathological states. Multiple histone deacetylase inhibitors have demonstrated neuroprotective efficacy in preclinical models of aging and neurodegenerative disorders [187], including sodium butyrate [188, 189], phenylbutyrate, valproate/valproic acid [190, 191], trichostatin A (TSA) [192, 193], suberoylanilide hydroxamic acid (SAHA) [194–196], Sirtuin 2 inhibitors. Other therapies that have been proven to alter disease progression, such as SIRT1 activator resveratrol [197], HAT activators [37], HMT inhibitors [198], metformin [187, 199–202], and rapamycin [16].(Table 2).

**Table 2:** Epigenetic therapeutics evaluated in preclinical models of neurodegenerative diseases.

PTM	Treatment/medicine	Targets and function	Disease	Result	References
Acetylation	Sodium Butyrate	HDACⅠ/Ⅱa (inhibitor)	AD	Attenuate cerebral Aβ burden during early disease stages in 5xFAD mice.	[188]
Acetylation	Sodium Butyrate	HDACⅠ/Ⅱa (inhibitor)	PD	Alleviate motor dysfunction and attenuate dopaminergic neuron loss in the striatum.	[189]
Acetylation	Valproic acid	HDACⅠ/Ⅱa (inhibitor)	AD	Ameliorate spatial memory impairment and Aβ deposition via the inhibition of inflammation.	[190]
Acetylation	Valproic acid	HDACⅠ/Ⅱa (inhibitor)	PD	Partially ameliorated the behavioral deficits in 6-OHDA-lesioned rats.	[191]
Acetylation	Trichostatin A(TSA)	HDACⅠ/Ⅱ(inhibitor)	AD	The intervention reduced brain levels of Aβ plaques and soluble Aβ oligomers.	[192]
Acetylation	Trichostatin A(TSA)	HDACⅠ/Ⅱ(inhibitor)	PD	Treatment attenuated MPTP-induced neuron death, concomitantly increasing BDNF gene expression and tyrosine hydroxylase in mice.	[193]
Acetylation	Trichostatin A(TSA), Sodium Butyrate	HDAC(inhibitor)	ALS/FTD	HDACi increases cellular survival and ameliorates TDP-43 toxicity.	[221]
Acetylation	Suberoylanilide hydroxamic acid(SAHA)	Pan HDAC (inhibitor)	AD	Attenuate neurobehavioral changes caused by Aβ injection.	[194]
Acetylation	Suberoylanilide hydroxamic acid(SAHA)	Pan HDAC (inhibitor)	PD	Mediating neuroprotection by inhibiting histone deacetylation to promote the release of neurotrophic factors by astrocytes.	[195]
Acetylation	Suberoylanilide hydroxamic acid(SAHA)	Pan HDAC (inhibitor)	HD	Improve the motor impairment in R6/2 mice and increased histone acetylation in the brain.	[196]
Acetylation	Resveratrol	SIRT1 (activator)	AD	Decreases CSF MMP9, modulates neuro-inflammation, and induces adaptive immunity.	[197]
Acetylation	ACY-738	HDAC6(inhibitor)	ALS/FTD	HDACi increases life span, survival rate and restores motor neuron phenotypes.	[167]
Methylation	UNC0642	HMT (G9a and GLP) (inhibitor)	AD	Ameliorate repressive chromatin marks such as H3K9me2, inhibit Aβ plaque formation, promote synaptic plasticity, and restore neuronal markers depleted in AD.	[198]
Methylation	ORY-2001	HMT(LSD1))(inhibitor)	AD	Mitigate cognitive impairment and reduce neuroinflammation, as well as normalize genes related to cognitive function, neuroplasticity and memory.	[222]
Methylation	BCI-121	HMT(Smyd3)(inhibitor)	AD	Improve the cognitive behavior of mice.	[91]
Methylation	A-366	HMT(EHMT1/2)(inhibitor)	PD	Restore synaptic damage and motor impairment in a PFF animal model.	[125]
Methylation	GSK-J4	HMT (KDM6A/B and KDM5B/C)(inhibitor)	PD	Upregulating H3K4me3 increased ferroportin-1 expression and neuroprotection.	[134]
Methylation	Rapamycin		Aging	Rapamycin ameliorates select age-associated histone modifications, including H3K18ac, H3K4me2 and H3K27me3.	[16]
	Metformin		AD	Reduce the expression of activated glial cells and pro-inflammatory factors. Activates AMPK and inhibits the mTOR signaling pathway, thereby enhancing the autophagy process and promoting the lysosomal degradation of Aβ.	[199, 201]
Phosphorylation	Metformin		PD	Alleviate MPP^+^-induced cytotoxicity and attenuated neuronal apoptosis. Prevent SNCA phosphorylation and aggregation and promote damaged mitochondrial clearance.	[200, 202]

Currently, most histone modification treatments for degenerative diseases are conducted at the animal experimental level [203]. Encouragingly, several deacetylase inhibitors have advanced to clinical development, with some achieving clinical application [204]. Notably, the pan-HDAC inhibitor vorinostat is undergoing Phase 1/2 clinical trials for AD, among other conditions including epilepsy and Crohn’s disease, with some achieving clinical application [204], [205]. HDAC6-targeted inhibitors, including ACY-1215 (licorinostat) and ACY-241 (sitanostat), which enhance α-tubulin acetylation and correct mutation-induced axonal transport defects, are currently in clinical trials [206, 207]. MC1568, a specific HDACIIa inhibitor, protects dopaminergic neurons in PD cell models from neurotoxins and α-synuclein-induced degeneration [208]. However, most available drugs are pan-HDAC inhibitors without specificity, and elevated global acetylation may result in off-target gene overexpression, potentially triggering detrimental effects [207]. The development of specific inhibitors requires further in-depth research in the future.

### Non-pharmacological interventions: lifestyle and diet management

Appropriate levels of physical activity and healthy dietary habits offer non-invasive and highly effective benefits, alleviating aging and neurodegenerative diseases through epigenetic modifications [209]. For example, aquatic exercise programs have been shown to improve the functionality of PD patients, particularly enhancing their mobility and functional capacity. Notably, after exercise interventions, levels of BDNF and histone H4 acetylation were found to increase [210]. Regular physical activity elevates systemic lactate levels, which cross the BBB to confer neuroprotective effects against neurodegenerative diseases [211].

Dietary restriction, including daily or intermittent caloric restriction (CR), reduces biomarkers associated with aging. Ketone bodies (KB) and ketogenic diets (KD) confer neuroprotection through coordinated modulation of cellular processes via metabolic and signaling mechanisms. These interventions attenuate apoptosis and oxidative stress, enhance mitochondrial function, and regulate PTMs in both histone and non-histone proteins [212]. Meanwhile, a six-month randomized controlled trial demonstrated that KB could improve the cognitive outcomes of patients with mild cognitive impairment [213]. This might be related to histone β-hydroxybutyrylation [214], but more research is still needed to reveal its epigenetic mechanism. Furthermore, KB/KD mitigate neuroinflammation while modulating autophagy, neurotransmission, and gut microbiome dynamics [215].

In addition, there are more novel methods for intervening in aging, including gene and cell therapy [216], [217], as well as reprogramming [218], [219], allogeneic chimerism, microbiota transplantation, immunotherapy, etc. These intervention measures offer promising approaches to slowing down cellular aging, extending healthspan and even overall lifespan [220].(Table 2).

## Conclusion

This review synthesizes the complex interplay between histone modifications and brain cell aging, elucidating their critical role in neurodegenerative pathologies including AD, PD, HD, ALS/FTD, and MS. Our analysis of the current literature reveals that post-translational histone modifications dynamically regulate gene expression and cellular functions in the aging brain. Specifically, histone marks that promote transcription at synaptic plasticity and memory-related gene promoters diminish with age, while repressive modifications accumulate. Consequently, the expression of these neuroprotective genes declines. Conversely, immune and inflammatory genes exhibit opposing epigenetic trajectories. Dysregulation of these mechanisms can trigger a cascade of cellular dysfunctions, ultimately contributing to age-related cognitive decline and neurodegenerative disease.

Given the reversible nature of these epigenetic changes, pharmacological interventions targeting histone modification states represent a promising novel strategy for addressing age-related cognitive and behavioral decline. Beyond established histone-modifying enzyme inhibitors and activators, environmental factors (such as diet, exercise, and stress) have been demonstrated to influence histone modification patterns, offering additional avenues for interventions promoting healthy brain aging. Elucidating the mechanisms underlying these epigenetic alterations and their interactions with genetic and environmental factors is critical for developing effective therapeutics. While research on histone acetylation has advanced to clinical trials, highlighting the translational potential of epigenetic therapy for brain aging and neurodegenerative disorders, investigations into lysine lactylation remain nascent and warrant extensive exploration to define its therapeutic relevance.

Comprehending the epigenetic mechanisms underpinning brain aging is critical for the development of targeted therapeutic strategies. These mechanisms demonstrate significant cell-type specificity among neurons, glia, and vascular cells, with each population exhibiting distinct epigenetic landscapes and responses to age-related stressors. Elucidating this cellular heterogeneity yields crucial insights into the diverse pathogenic mechanisms underlying age-related neurodegenerative disorders. Critically, elucidating these molecular pathways facilitates earlier disease detection, underpins preventive strategies to counteract age-related pathological processes, and directs the development of enhanced therapeutic interventions. Ultimately, delaying the progression of brain aging maintains cognitive faculties, memory retention, and functional independence in the aging population, thereby enhancing quality of life.

## References

[1] Cai Y, Song W, Li J, Jing Y, Liang C, Zhang L, et al. The landscape of aging. Sci China Life Sci. 2022;65:2354–454. 36066811 10.1007/s11427-022-2161-3 PMC9446657

[2] Nyberg L, Wåhlin A. The many facets of brain aging. eLife. 2020;9:e56640. 10.7554/eLife.56640 PMC7162651 32297862

[3] Mattson MP, Arumugam TV. Hallmarks of brain aging: adaptive and pathological modification by metabolic states. Cell Metab. 2018;27:1176–99. 29874566 10.1016/j.cmet.2018.05.011 PMC6039826

[4] Melo Dos Santos LS, Trombetta-Lima M, Eggen B, Demaria M. Cellular senescence in brain aging and neurodegeneration. Ageing Res Rev. 2024;93:102141. 38030088 10.1016/j.arr.2023.102141

[5] Sikora E, Bielak-Zmijewska A, Dudkowska M, Krzystyniak A, Mosieniak G, Wesierska M, et al. Cellular senescence in brain aging. Front Aging Neurosci. 2021;13:646924. 33732142 10.3389/fnagi.2021.646924 PMC7959760

[6] Salas IH, Burgado J, Allen NJ. Glia: victims or villains of the aging brain? Neurobiol Dis. 2020;143:105008. 32622920 10.1016/j.nbd.2020.105008

[7] Lupo G, Gaetani S, Cacci E, Biagioni S, Negri R. Molecular signatures of the aging brain: finding the links between genes and phenotypes. NeuroTherapeutics. 2019;16:543–53. 31161490 10.1007/s13311-019-00743-2 PMC6694319

[8] Soraci L, Corsonello A, Paparazzo E, Montesanto A, Piacenza F, Olivieri F, et al. Neuroinflammaging: a tight line between normal aging and age-related neurodegenerative disorders. Aging Dis. 2024;15:1726–47. 38300639 10.14336/AD.2023.1001 PMC11272206

[9] Coupé P, Manjón JV, Lanuza E, Catheline G. Lifespan changes of the human brain in Alzheimer’s disease. Sci Rep. 2019;9:3998. 30850617 10.1038/s41598-019-39809-8 PMC6408544

[10] Kubben N, Misteli T. Shared molecular and cellular mechanisms of premature ageing and ageing-associated diseases. Nat Rev Mol Cell Biol. 2017;18:595–609. 28792007 10.1038/nrm.2017.68 PMC6290461

[11] Song S, Tchkonia T, Jiang J, Kirkland JL, Sun Y. Targeting senescent cells for a healthier aging: challenges and opportunities. Adv Sci. 2020;7:2002611. 10.1002/advs.202002611 PMC7709980 33304768

[12] Strahl BD, Allis CD. The language of covalent histone modifications. Nature. 2000;403:41–5. 10638745 10.1038/47412

[13] Suganuma T, Workman JL. Crosstalk among histone modifications. Cell. 2008;135:604–7. 19013272 10.1016/j.cell.2008.10.036

[14] Stoccoro A, Coppedè F. Role of epigenetics in Alzheimer’s disease pathogenesis. Neurodegener Dis Manag. 2018;8:181–93. 29888987 10.2217/nmt-2018-0004

[15] Wu Z, Zhang W, Qu J, Liu GH. Emerging epigenetic insights into aging mechanisms and interventions. Trends Pharmacol Sci. 2024;45:157–72. 38216430 10.1016/j.tips.2023.12.002

[16] Gong H, Qian H, Ertl R, Astle CM, Wang GG, Harrison DE, et al. Histone modifications change with age, dietary restriction and rapamycin treatment in mouse brain. Oncotarget. 2015;6:15882–90. 26021816 10.18632/oncotarget.4137 PMC4599244

[17] Sidler C, Kovalchuk O, Kovalchuk I. Epigenetic regulation of cellular senescence and aging. Front Genet. 2017;8:138. 29018479 10.3389/fgene.2017.00138 PMC5622920

[18] Bassi S, Tripathi T, Monziani A, Di Leva F, Biagioli M. Epigenetics of Huntington’s disease. Adv Exp Med Biol. 2017;978:277–99. 28523552 10.1007/978-3-319-53889-1_15

[19] Tai HC, Schuman EM. Ubiquitin, the proteasome and protein degradation in neuronal function and dysfunction. Nat Rev Neurosci. 2008;9:826–38. 18931696 10.1038/nrn2499

[20] Schmidt MF, Gan ZY, Komander D, Dewson G. Ubiquitin signalling in neurodegeneration: mechanisms and therapeutic opportunities. Cell Death Differ. 2021;28:570–90. 33414510 10.1038/s41418-020-00706-7 PMC7862249

[21] Song L, Rape M. Reverse the curse-the role of deubiquitination in cell cycle control. Curr Opin Cell Biol. 2008;20:156–63. 18346885 10.1016/j.ceb.2008.01.012 PMC2387050

[22] Koyuncu S, Loureiro R, Lee HJ, Wagle P, Krueger M, Vilchez D. Rewiring of the ubiquitinated proteome determines ageing in C. elegans. Nature. 2021;596:285–90. 34321666 10.1038/s41586-021-03781-z PMC8357631

[23] Yang L, Ma Z, Wang H, Niu K, Cao Y, Sun L, et al. Ubiquitylome study identifies increased histone 2A ubiquitylation as an evolutionarily conserved aging biomarker. Nat Commun. 2019;10:2191. 31113955 10.1038/s41467-019-10136-w PMC6529468

[24] Kelmer Sacramento E, Kirkpatrick JM, Mazzetto M, Baumgart M, Bartolome A, Di Sanzo S, et al. Reduced proteasome activity in the aging brain results in ribosome stoichiometry loss and aggregation. Mol Syst Biol. 2020;16:e9596. 32558274 10.15252/msb.20209596 PMC7301280

[25] Wang L, Sooram B, Kumar R, Schedin-Weiss S, Tjernberg LO, Winblad B. Tau degradation in Alzheimer’s disease: mechanisms and therapeutic opportunities. Alzheimer’s Dement. 2025;21. e70048. 40109019 10.1002/alz.70048 PMC11923393

[26] Rossetto D, Avvakumov N, Côté J. Histone phosphorylation: a chromatin modification involved in diverse nuclear events. Epigenetics. 2012;7:1098–108. 22948226 10.4161/epi.21975 PMC3469451

[27] Sedelnikova OA, Horikawa I, Zimonjic DB, Popescu NC, Bonner WM, Barrett JC. Senescing human cells and ageing mice accumulate DNA lesions with unrepairable double-strand breaks. Nat Cell Biol. 2004;6:168–70. 14755273 10.1038/ncb1095

[28] Wu ZX, Cao L, Li XW, Jiang W, Li XY, Xu J, et al. Accelerated deficits of spatial learning and memory resulting from prenatal inflammatory insult are correlated with abnormal phosphorylation and methylation of histone 3 in CD-1 Mice. Front Aging Neurosci. 2019;11:114. 31156421 10.3389/fnagi.2019.00114 PMC6531990

[29] Zhang D, Tang Z, Huang H, Zhou G, Cui C, Weng Y, et al. Metabolic regulation of gene expression by histone lactylation. Nature. 2019;574:575–80. 31645732 10.1038/s41586-019-1678-1 PMC6818755

[30] Dai X, Lv X, Thompson EW, Ostrikov KK. Histone lactylation: epigenetic mark of glycolytic switch. Trends Genet. 2022;38:124–7. 34627643 10.1016/j.tig.2021.09.009

[31] Moreno-Yruela C, Zhang D, Wei W, Bæk M, Liu W, Gao J, et al. Class I histone deacetylases (HDAC1-3) are histone lysine delactylases. Sci Adv. 2022;8:eabi6696. 35044827 10.1126/sciadv.abi6696 PMC8769552

[32] Wang Y, Li P, Xu Y, Feng L, Fang Y, Song G, et al. Lactate metabolism and histone lactylation in the central nervous system disorders: impacts and molecular mechanisms. J Neuroinflamm. 2024;21:308. 10.1186/s12974-024-03303-4 PMC11605911 39609834

[33] Pan RY, He L, Zhang J, Liu X, Liao Y, Gao J, et al. Positive feedback regulation of microglial glucose metabolism by histone H4 lysine 12 lactylation in Alzheimer’s disease. Cell Metab. 2022;34:634–648.e636. 35303422 10.1016/j.cmet.2022.02.013

[34] Banks WA, Reed MJ, Logsdon AF, Rhea EM, Erickson MA. Healthy aging and the blood-brain barrier. Nat aging. 2021;1:243–54. 34368785 10.1038/s43587-021-00043-5 PMC8340949

[35] Zhang W, Qu J, Liu GH, Belmonte JCI. The ageing epigenome and its rejuvenation. Nat Rev Mol Cell Biol. 2020;21:137–50. 32020082 10.1038/s41580-019-0204-5

[36] Kandlur A, Satyamoorthy K, Gangadharan G. Oxidative stress in cognitive and epigenetic aging: a retrospective glance. Front Mol Neurosci. 2020;13:41. 32256315 10.3389/fnmol.2020.00041 PMC7093495

[37] Neal M, Richardson JR. Epigenetic regulation of astrocyte function in neuroinflammation and neurodegeneration. Biochim et Biophys Acta Mol Basis Dis. 2018;1864:432–43. 10.1016/j.bbadis.2017.11.004 PMC5743548 29113750

[38] Budamagunta V, Kumar A, Rani A, Bean L, Manohar-Sindhu S, Yang Y, et al. Effect of peripheral cellular senescence on brain aging and cognitive decline. Aging cell. 2023;22:e13817. 36959691 10.1111/acel.13817 PMC10186609

[39] Song S, Johnson FB. Epigenetic mechanisms impacting aging: a focus on histone levels and telomeres. Genes 2018;9:201. 10.3390/genes9040201 PMC5924543 29642537

[40] Cheung P, Vallania F, Warsinske HC, Donato M, Schaffert S, Chang SE, et al. Single-cell chromatin modification profiling reveals increased epigenetic variations with aging. Cell. 2018;173:1385–97.e1314. 29706550 10.1016/j.cell.2018.03.079 PMC5984186

[41] Kishimoto S, Uno M, Okabe E, Nono M, Nishida E. Environmental stresses induce transgenerationally inheritable survival advantages via germline-to-soma communication in Caenorhabditis elegans. Nat Commun. 2017;8:14031. 28067237 10.1038/ncomms14031 PMC5227915

[42] Barter JD, Foster TC. Aging in the brain: new roles of epigenetics in cognitive decline. Neuroscientist. 2018;24:516–25. 29877135 10.1177/1073858418780971

[43] Cheng H, Xuan H, Green CD, Han Y, Sun N, Shen H, et al. Repression of human and mouse brain inflammaging transcriptome by broad gene-body histone hyperacetylation. Proc Natl Acad Sci USA. 2018;115:7611–6. 29967166 10.1073/pnas.1800656115 PMC6055154

[44] Singh P, Thakur MK. Histone Deacetylase 2 inhibition attenuates downregulation of hippocampal plasticity gene expression during aging. Mol Neurobiol. 2018;55:2432–42. 28364391 10.1007/s12035-017-0490-x

[45] Grabowska W, Sikora E, Bielak-Zmijewska A. Sirtuins, a promising target in slowing down the ageing process. Biogerontology. 2017;18:447–76. 28258519 10.1007/s10522-017-9685-9 PMC5514220

[46] Fang EF, Lautrup S, Hou Y, Demarest TG, Croteau DL, Mattson MP, et al. NAD(+) in aging: molecular mechanisms and translational implications. Trends Mol Med. 2017;23:899–916. 28899755 10.1016/j.molmed.2017.08.001 PMC7494058

[47] Braidy N, Poljak A, Grant R, Jayasena T, Mansour H, Chan-Ling T, et al. Differential expression of sirtuins in the aging rat brain. Front Cell Neurosci. 2015;9:167. 26005404 10.3389/fncel.2015.00167 PMC4424846

[48] Gao J, Wang WY, Mao YW, Gräff J, Guan JS, Pan L, et al. A novel pathway regulates memory and plasticity via SIRT1 and miR-134. Nature. 2010;466:1105–9. 20622856 10.1038/nature09271 PMC2928875

[49] Ng F, Wijaya L, Tang BL. SIRT1 in the brain-connections with aging-associated disorders and lifespan. Front Cell Neurosci. 2015;9:64. 25805970 10.3389/fncel.2015.00064 PMC4353374

[50] Palomer E, Martín-Segura A, Baliyan S, Ahmed T, Balschun D, Venero C, et al. Aging triggers a repressive chromatin state at bdnf promoters in hippocampal neurons. Cell Rep. 2016;16:2889–900. 27626660 10.1016/j.celrep.2016.08.028

[51] Santos-Rosa H, Schneider R, Bannister AJ, Sherriff J, Bernstein BE, Emre NC, et al. Active genes are tri-methylated at K4 of histone H3. Nature. 2002;419:407–11. 12353038 10.1038/nature01080

[52] Collins BE, Sweatt JD, Greer CB. Broad domains of histone 3 lysine 4 trimethylation are associated with transcriptional activation in CA1 neurons of the hippocampus during memory formation. Neurobiol Learn Mem. 2019;161:149–57. 31002880 10.1016/j.nlm.2019.04.009 PMC6541021

[53] Cheung I, Shulha HP, Jiang Y, Matevossian A, Wang J, Weng Z, et al. Developmental regulation and individual differences of neuronal H3K4me3 epigenomes in the prefrontal cortex. Proc Natl Acad Sci USA. 2010;107:8824–9. 20421462 10.1073/pnas.1001702107 PMC2889328

[54] Franceschi C, Garagnani P, Morsiani C, Conte M, Santoro A, Grignolio A, et al. The continuum of aging and age-related diseases: common mechanisms but different rates. Front Med. 2018;5:61. 10.3389/fmed.2018.00061 PMC5890129 29662881

[55] Yang N, Sen P. The senescent cell epigenome. Aging. 2018;10:3590–609. 30391936 10.18632/aging.101617 PMC6286853

[56] Kushwaha A, Thakur MK. Increase in hippocampal histone H3K9me3 is negatively correlated with memory in old male mice. Biogerontology. 2020;21:175–89. 31760560 10.1007/s10522-019-09850-1

[57] Kushwaha A, Thakur MK. Suv39h1 silencing recovers memory decline in scopolamine-induced amnesic mouse model. Mol Neurobiol. 2024;61:487–97. 37626270 10.1007/s12035-023-03570-x

[58] Yuan J, Chang SY, Yin SG, Liu ZY, Cheng X, Liu XJ, et al. Two conserved epigenetic regulators prevent healthy ageing. Nature. 2020;579:118–22. 32103178 10.1038/s41586-020-2037-y

[59] Jiang X, Yang Y, Li X, Li T, Yu T, Fu X. Lactylation: an innovative approach to disease control. Aging Dis. 2024;16:2130–50. 39325940 10.14336/AD.2024.0918 PMC12221411

[60] Wu Y, Hu H, Liu W, Zhao Y, Xie F, Sun Z, et al. Hippocampal lactate-infusion enhances spatial memory correlated with monocarboxylate transporter 2 and Lactylation. Brain Sci. 2024;14:327. 10.3390/brainsci14040327 PMC11048250 38671979

[61] Lei Z, Mozaffaritabar S, Kawamura T, Koike A, Kolonics A, Kéringer J, et al. The effects of long-term lactate and high-intensity interval training (HIIT) on brain neuroplasticity of aged mice. Heliyon. 2024;10:e24421. 38293399 10.1016/j.heliyon.2024.e24421 PMC10826720

[62] Ross JM, Öberg J, Brené S, Coppotelli G, Terzioglu M, Pernold K, et al. High brain lactate is a hallmark of aging and caused by a shift in the lactate dehydrogenase A/B ratio. Proc Natl Acad Sci USA. 2010;107:20087–92. 21041631 10.1073/pnas.1008189107 PMC2993405

[63] Quistorff B, Grunnet N. High brain lactate is not caused by a shift in the lactate dehydrogenase A/B ratio. Proc Natl Acad Sci USA. 2011;108:E21. 21278331 10.1073/pnas.1017750108 PMC3041136

[64] Costa J, Martins S, Ferreira PA, Cardoso AMS, Guedes JR, Peça J, et al. The old guard: Age-related changes in microglia and their consequences. Mech Ageing Dev. 2021;197:111512. 34022277 10.1016/j.mad.2021.111512

[65] Auzmendi-Iriarte J, Moreno-Cugnon L, Saenz-Antoñanzas A, Grassi D, de Pancorbo MM, Arevalo MA, et al. High levels of HDAC expression correlate with microglial aging. Expert Opin Ther Targets. 2022;26:911–22. 10.1080/14728222.2022.2158081 36503367

[66] Cornejo F, von Bernhardi R. Age-dependent changes in the activation and regulation of microglia. Adv Exp Med Biol. 2016;949:205–26. 27714691 10.1007/978-3-319-40764-7_10

[67] Tang Y, Li T, Li J, Yang J, Liu H, Zhang XJ, et al. Jmjd3 is essential for the epigenetic modulation of microglia phenotypes in the immune pathogenesis of Parkinson’s disease. Cell Death Differ. 2014;21:369–80. 24212761 10.1038/cdd.2013.159 PMC3921590

[68] von Bernhardi R, Eugenín-von Bernhardi L, Eugenín J. Microglial cell dysregulation in brain aging and neurodegeneration. Front Aging Neurosci. 2015;7:124. 26257642 10.3389/fnagi.2015.00124 PMC4507468

[69] Hartmann C, Haß C, Knobloch M, Barrantes I, Fumagalli L, Premereur J, et al. Prematurely aged human microglia exhibit impaired stress response and defective nucleocytoplasmic shuttling of ALS Associated FUS. Aging Cell. 2025;24:e70232. 40970514 10.1111/acel.70232 PMC12610945

[70] Škandík M, Friess L, Vázquez-Cabrera G, Keane L, Grabert K, Cruz De Los Santos M, et al. Age-associated microglial transcriptome leads to diminished immunogenicity and dysregulation of MCT4 and P2RY12/P2RY13 related functions. Cell Death Discov. 2025;11:16. 39828750 10.1038/s41420-025-02295-1 PMC11743796

[71] Zhang X, Wang Y, Yuan J, Li N, Pei S, Xu J, et al. Macrophage/microglial Ezh2 facilitates autoimmune inflammation through inhibition of Socs3. J Exp Med. 2018;215:1365–82. 29626115 10.1084/jem.20171417 PMC5940261

[72] Faraco G, Pittelli M, Cavone L, Fossati S, Porcu M, Mascagni P, et al. Histone deacetylase (HDAC) inhibitors reduce the glial inflammatory response in vitro and in vivo. Neurobiol Dis. 2009;36:269–79. 19635561 10.1016/j.nbd.2009.07.019

[73] Cui W, Allen ND, Skynner M, Gusterson B, Clark AJ. Inducible ablation of astrocytes shows that these cells are required for neuronal survival in the adult brain. Glia. 2001;34:272–82. 11360300 10.1002/glia.1061

[74] Adamu A, Li S, Gao F, Xue G. The role of neuroinflammation in neurodegenerative diseases: current understanding and future therapeutic targets. Front Aging Neurosci. 2024;16:1347987. 38681666 10.3389/fnagi.2024.1347987 PMC11045904

[75] Kalinin S, Polak PE, Lin SX, Braun D, Guizzetti M, Zhang X, et al. Dimethyl fumarate regulates histone deacetylase expression in astrocytes. J Neuroimmunol. 2013;263:13–9. 23916696 10.1016/j.jneuroim.2013.07.007

[76] Correa F, Mallard C, Nilsson M, Sandberg M. Activated microglia decrease histone acetylation and Nrf2-inducible anti-oxidant defence in astrocytes: restoring effects of inhibitors of HDACs, p38 MAPK and GSK3β. Neurobiol Dis. 2011;44:142–51. 21757005 10.1016/j.nbd.2011.06.016 PMC3341174

[77] Marinova Z, Leng Y, Leeds P, Chuang DM. Histone deacetylase inhibition alters histone methylation associated with heat shock protein 70 promoter modifications in astrocytes and neurons. Neuropharmacology. 2011;60:1109–15. 20888352 10.1016/j.neuropharm.2010.09.022 PMC3036778

[78] Montagne A, Barnes SR, Sweeney MD, Halliday MR, Sagare AP, Zhao Z, et al. Blood-brain barrier breakdown in the aging human hippocampus. Neuron. 2015;85:296–302. 25611508 10.1016/j.neuron.2014.12.032 PMC4350773

[79] Cunha S, Bicker J, Sereno J, Falcão A, Fortuna A. Blood brain barrier dysfunction in healthy aging and dementia: why, how, what for? Ageing Res Rev. 2024;99:102395. 38950867 10.1016/j.arr.2024.102395

[80] Knox EG, Aburto MR, Clarke G, Cryan JF, O’Driscoll CM. The blood-brain barrier in aging and neurodegeneration. Mol Psychiatry. 2022;27:2659–73. 35361905 10.1038/s41380-022-01511-z PMC9156404

[81] Stamatovic SM, Martinez-Revollar G, Hu A, Choi J, Keep RF, Andjelkovic AV. Decline in Sirtuin-1 expression and activity plays a critical role in blood-brain barrier permeability in aging. Neurobiol Dis. 2019;126:105–16. 30196051 10.1016/j.nbd.2018.09.006 PMC6401345

[82] Li Y, Di C, Song S, Zhang Y, Lu Y, Liao J, et al. Choroid plexus mast cells drive tumor-associated hydrocephalus. Cell. 2023;186:5719–38.e5728. 38056463 10.1016/j.cell.2023.11.001

[83] Lu T, Aron L, Zullo J, Pan Y, Kim H, Chen Y, et al. REST and stress resistance in ageing and Alzheimer’s disease. Nature. 2014;507:448–54. 24670762 10.1038/nature13163 PMC4110979

[84] 2023 Alzheimer’s disease facts and figures. Alzheimer’s Dement. 2023;19:1598–695. 10.1002/alz.13016 36918389

[85] Lin MT, Beal MF. Mitochondrial dysfunction and oxidative stress in neurodegenerative diseases. Nature. 2006;443:787–95. 17051205 10.1038/nature05292

[86] Beaver M, Karisetty BC, Zhang H, Bhatnagar A, Armour E, Parmar V, et al. Chromatin and transcriptomic profiling uncover dysregulation of the Tip60 HAT/HDAC2 epigenomic landscape in the neurodegenerative brain. Epigenetics. 2022;17:786–807. 34369292 10.1080/15592294.2021.1959742 PMC9336495

[87] Singh P, Thakur MK. Reduced recognition memory is correlated with decrease in DNA methyltransferase1 and increase in histone deacetylase2 protein expression in old male mice. Biogerontology. 2014;15:339–46. 24924148 10.1007/s10522-014-9504-5

[88] Lin Y, Lin A, Cai L, Huang W, Yan S, Wei Y, et al. ACSS2-dependent histone acetylation improves cognition in mouse model of Alzheimer’s disease. Mol Neurodegener. 2023;18:47. 37438762 10.1186/s13024-023-00625-4 PMC10339567

[89] Wang W, Cao Q, Tan T, Yang F, Williams JB, Yan Z. Epigenetic treatment of behavioral and physiological deficits in a tauopathy mouse model. Aging Cell. 2021;20:e13456. 34547169 10.1111/acel.13456 PMC8520711

[90] Zheng Y, Liu A, Wang ZJ, Cao Q, Wang W, Lin L, et al. Inhibition of EHMT1/2 rescues synaptic and cognitive functions for Alzheimer’s disease. Brain. 2019;142:787–807. 30668640 10.1093/brain/awy354 PMC6391616

[91] Williams JB, Cao Q, Wang W, Lee YH, Qin L, Zhong P, et al. Inhibition of histone methyltransferase Smyd3 rescues NMDAR and cognitive deficits in a tauopathy mouse model. Nat Commun. 2023;14:91. 36609445 10.1038/s41467-022-35749-6 PMC9822922

[92] Klein HU, McCabe C, Gjoneska E, Sullivan SE, Kaskow BJ, Tang A, et al. Epigenome-wide study uncovers large-scale changes in histone acetylation driven by tau pathology in aging and Alzheimer’s human brains. Nat Neurosci. 2019;22:37–46. 30559478 10.1038/s41593-018-0291-1 PMC6516529

[93] Bryan Iii MR, Tian X, Tseng JH, Evangelista BA, Ragusa JV, Bryan AF, et al. Development and characterization of novel anti-acetylated tau monoclonal antibodies to probe pathogenic tau species in Alzheimer’s disease. Acta Neuropathol Commun. 2024;12:163. 39396065 10.1186/s40478-024-01865-1 PMC11470691

[94] Tseng JH, Xie L, Song S, Xie Y, Allen L, Ajit D, et al. The deacetylase HDAC6 mediates endogenous neuritic Tau pathology. Cell Rep. 2017;20:2169–83. 28854366 10.1016/j.celrep.2017.07.082 PMC5578720

[95] Choi H, Kim HJ, Yang J, Chae S, Lee W, Chung S, et al. Acetylation changes tau interactome to degrade tau in Alzheimer’s disease animal and organoid models. Aging Cell. 2020;19:e13081. 31763743 10.1111/acel.13081 PMC6974726

[96] Caballero B, Bourdenx M, Luengo E, Diaz A, Sohn PD, Chen X, et al. Acetylated tau inhibits chaperone-mediated autophagy and promotes tau pathology propagation in mice. Nat Commun. 2021;12:2238. 33854069 10.1038/s41467-021-22501-9 PMC8047017

[97] Trzeciakiewicz H, Ajit D, Tseng JH, Chen Y, Ajit A, Tabassum Z, et al. An HDAC6-dependent surveillance mechanism suppresses tau-mediated neurodegeneration and cognitive decline. Nat Commun. 2020;11:5522. 33139698 10.1038/s41467-020-19317-4 PMC7606452

[98] An X, He J, Xie P, Li C, Xia M, Guo D, et al. The effect of tau K677 lactylation on ferritinophagy and ferroptosis in Alzheimer’s disease. Free Radic Biol Med. 2024;224:685–706. 39307193 10.1016/j.freeradbiomed.2024.09.021

[99] Nativio R, Lan Y, Donahue G, Sidoli S, Berson A, Srinivasan AR, et al. An integrated multi-omics approach identifies epigenetic alterations associated with Alzheimer’s disease. Nat Genet. 2020;52:1024–35. 32989324 10.1038/s41588-020-0696-0 PMC8098004

[100] Xu DC, Sas-Nowosielska H, Donahue G, Huang H, Pourshafie N, Good CR, et al. Histone acetylation in an Alzheimer’s disease cell model promotes homeostatic amyloid-reducing pathways. Acta Neuropathol Commun. 2024;12:3. 38167174 10.1186/s40478-023-01696-6 PMC10759377

[101] Marzi SJ, Leung SK, Ribarska T, Hannon E, Smith AR, Pishva E, et al. A histone acetylome-wide association study of Alzheimer’s disease identifies disease-associated H3K27ac differences in the entorhinal cortex. Nat Neurosci. 2018;21:1618–27. 30349106 10.1038/s41593-018-0253-7

[102] Li N, Bai N, Zhao X, Cheng R, Wu X, Jiang B, et al. Cooperative effects of SIRT1 and SIRT2 on APP acetylation. Aging Cell. 2023;22:e13967. 37602729 10.1111/acel.13967 PMC10577574

[103] Bai N, Li N, Cheng R, Guan Y, Zhao X, Song Z, et al. Inhibition of SIRT2 promotes APP acetylation and ameliorates cognitive impairment in APP/PS1 transgenic mice. Cell Rep. 2022;40:111062. 35830807 10.1016/j.celrep.2022.111062

[104] Cheng R, Bai N, Liu S, Zhao X, Jiang B, Guo W, et al. The deacetylase SIRT6 reduces amyloid pathology and supports cognition in mice by reducing the stability of APP in neurons. Sci Signal. 2024;17:eado1035. 39656860 10.1126/scisignal.ado1035

[105] Perone I, Ghena N, Wang J, Mackey C, Wan R, Malla S, et al. Mitochondrial SIRT3 deficiency results in neuronal network hyperexcitability, accelerates age-related Aβ pathology, and renders neurons vulnerable to Aβ toxicity. Neuromol. Med. 2023;25:27–39. 10.1007/s12017-022-08713-2 PMC9810471 35749057

[106] Datta M, Staszewski O, Raschi E, Frosch M, Hagemeyer N, Tay TL, et al. Histone Deacetylases 1 and 2 regulate microglia function during development, homeostasis, and neurodegeneration in a context-dependent manner. Immunity. 2018;48:514–29.e516. 29548672 10.1016/j.immuni.2018.02.016

[107] Sun XY, Zheng T, Yang X, Liu L, Gao SS, Xu HB, et al. HDAC2 hyperexpression alters hippocampal neuronal transcription and microglial activity in neuroinflammation-induced cognitive dysfunction. J Neuroinflamm. 2019;16:249. 10.1186/s12974-019-1640-z PMC6889553 31796106

[108] Zhu X, Wang S, Yu L, Jin J, Ye X, Liu Y, et al. HDAC3 negatively regulates spatial memory in a mouse model of Alzheimer’s disease. Aging Cell. 2017;16:1073–82. 28771976 10.1111/acel.12642 PMC5595690

[109] Cheng J, Zhao H. NEK7 induces lactylation in Alzheimer’s disease to promote pyroptosis in BV-2 cells. Mol Brain. 2024;17:81. 39563448 10.1186/s13041-024-01156-9 PMC11577724

[110] Wei L, Yang X, Wang J, Wang Z, Wang Q, Ding Y, et al. H3K18 lactylation of senescent microglia potentiates brain aging and Alzheimer’s disease through the NFκB signaling pathway. J Neuroinflamm. 2023;20:208. 10.1186/s12974-023-02879-7 PMC10494370 37697347

[111] Han H, Zhao Y, Du J, Wang S, Yang X, Li W, et al. Exercise improves cognitive dysfunction and neuroinflammation in mice through Histone H3 lactylation in microglia. Immun Ageing. 2023;20:63. 37978517 10.1186/s12979-023-00390-4 PMC10655345

[112] Anderson KW, Turko IV. Histone post-translational modifications in frontal cortex from human donors with Alzheimer’s disease. Clin Proteom. 2015;12:26. 10.1186/s12014-015-9098-1 PMC4591557 26435705

[113] Anderson KW, Mast N, Pikuleva IA, Turko IV. Histone H3 Ser57 and Thr58 phosphorylation in the brain of 5XFAD mice. FEBS Open Bio. 2015;5:550–6. 26199864 10.1016/j.fob.2015.06.009 PMC4506931

[114] Myung NH, Zhu X, Kruman II, Castellani RJ, Petersen RB, Siedlak SL, et al. Evidence of DNA damage in Alzheimer disease: phosphorylation of histone H2AX in astrocytes. Age. 2008;30:209–15. 19424844 10.1007/s11357-008-9050-7 PMC2585649

[115] Gibb WR, Lees AJ. The relevance of the Lewy body to the pathogenesis of idiopathic Parkinson’s disease. J Neurol Neurosurg Psychiatry. 1988;51:745–52. 2841426 10.1136/jnnp.51.6.745 PMC1033142

[116] Church FC. Treatment options for motor and non-motor symptoms of Parkinson’s Disease. Biomolecules 2021;11:612. 10.3390/biom11040612 PMC8074325 33924103

[117] Cacabelos R. Parkinson’s disease: from pathogenesis to pharmacogenomics. Int J Mol Sci. 2017;18:551. 10.3390/ijms18030551 PMC5372567 28273839

[118] Chen Y, Jiang Y, Yang Y, Huang X, Sun C. SIRT1 protects dopaminergic neurons in Parkinson’s disease models via PGC-1α-mediated mitochondrial biogenesis. Neurotox Res. 2021;39:1393–404. 34251648 10.1007/s12640-021-00392-4

[119] Shi H, Deng HX, Gius D, Schumacker PT, Surmeier DJ, Ma YC. Sirt3 protects dopaminergic neurons from mitochondrial oxidative stress. Hum Mol Genet. 2017;26:1915–26. 28369333 10.1093/hmg/ddx100 PMC6075394

[120] Toker L, Tran GT, Sundaresan J, Tysnes OB, Alves G, Haugarvoll K, et al. Genome-wide histone acetylation analysis reveals altered transcriptional regulation in the Parkinson’s disease brain. Mol Neurodegener. 2021;16:31. 33947435 10.1186/s13024-021-00450-7 PMC8097820

[121] Huang M, Jin H, Anantharam V, Kanthasamy A, Kanthasamy AG. Mitochondrial stress-induced H4K12 hyperacetylation dysregulates transcription in Parkinson’s disease. Front Cell Neurosci. 2024;18:1422362. 39188570 10.3389/fncel.2024.1422362 PMC11345260

[122] Harrison IF, Smith AD, Dexter DT. Pathological histone acetylation in Parkinson’s disease: neuroprotection and inhibition of microglial activation through SIRT 2 inhibition. Neurosci Lett. 2018;666:48–57. 29273397 10.1016/j.neulet.2017.12.037 PMC5821898

[123] Wu X, Chen PS, Dallas S, Wilson B, Block ML, Wang CC, et al. Histone deacetylase inhibitors up-regulate astrocyte GDNF and BDNF gene transcription and protect dopaminergic neurons. Int J Neuropsychopharmacol. 2008;11:1123–34. 18611290 10.1017/S1461145708009024 PMC2579941

[124] Huang M, Malovic E, Ealy A, Jin H, Anantharam V, Kanthasamy A, et al. Microglial immune regulation by epigenetic reprogramming through histone H3K27 acetylation in neuroinflammation. Front Immunol. 2023;14:1052925. 37033967 10.3389/fimmu.2023.1052925 PMC10073546

[125] Zhang Z, Wang R, Zhou H, Wu D, Cao Y, Zhang C, et al. Inhibition of EHMT1/2 rescues synaptic damage and motor impairment in a PD mouse model. Cell Mol Life Sci. 2024;81:128. 38472451 10.1007/s00018-024-05176-5 PMC10933175

[126] Outeiro TF, Kontopoulos E, Altmann SM, Kufareva I, Strathearn KE, Amore AM, et al. Sirtuin 2 inhibitors rescue alpha-synuclein-mediated toxicity in models of Parkinson’s disease. Science. 2007;317:516–9. 17588900 10.1126/science.1143780

[127] Li X, Liu T, Wu TT, Feng Y, Peng SJ, Yin H, et al. SIRT1 deacetylates TET2 and promotes its ubiquitination degradation to achieve neuroprotection against Parkinson’s disease. Front Neurol. 2021;12:652882. 33935952 10.3389/fneur.2021.652882 PMC8082066

[128] Chu Y, Hirst WD, Federoff HJ, Harms AS, Stoessl AJ, Kordower JH. Nigrostriatal tau pathology in Parkinsonism and Parkinson’s disease. Brain. 2024;147:444–57. 38006313 10.1093/brain/awad388 PMC10834249

[129] Wills J, Jones J, Haggerty T, Duka V, Joyce JN, Sidhu A. Elevated tauopathy and alpha-synuclein pathology in postmortem Parkinson’s disease brains with and without dementia. Exp Neurol. 2010;225:210–8. 20599975 10.1016/j.expneurol.2010.06.017 PMC2922478

[130] Roy B, Jackson GR. Interactions between Tau and α-synuclein augment neurotoxicity in a Drosophila model of Parkinson’s disease. Hum Mol Genet. 2014;23:3008–23. 24430504 10.1093/hmg/ddu011 PMC4014195

[131] Pan L, Li C, Meng L, Tian Y, He M, Yuan X, et al. Tau accelerates α-synuclein aggregation and spreading in Parkinson’s disease. Brain. 2022;145:3454–71. 35552614 10.1093/brain/awac171

[132] Esteves AR, Palma AM, Gomes R, Santos D, Silva DF, Cardoso SM. Acetylation as a major determinant to microtubule-dependent autophagy: relevance to Alzheimer’s and Parkinson disease pathology. Biochim et Biophys Acta Mol Basis Dis. 2019;1865:2008–23. 10.1016/j.bbadis.2018.11.014 30572013

[133] Zhang L, Zhou T, Su Y, He L, Wang Z. Involvement of histone methylation in the regulation of neuronal death. J Physiol Biochem. 2023;79:685–93. 37544979 10.1007/s13105-023-00978-w

[134] Mu MD, Qian ZM, Yang SX, Rong KL, Yung WH, Ke Y. Therapeutic effect of a histone demethylase inhibitor in Parkinson’s disease. Cell Death Dis. 2020;11:927. 33116116 10.1038/s41419-020-03105-5 PMC7595123

[135] Lan T, Hu L, Sun T, Wang X, Xiao Z, Shen D, et al. H3K9 trimethylation dictates neuronal ferroptosis through repressing Tfr1. J Cereb Blood Flow Metab. 2023;43:1365–81. 36960698 10.1177/0271678X231165653 PMC10369154

[136] MacDonald ME, Ambrose CM, Duyao MP, Myers RH, Lin C, Srinidhi L, Barnes G, Taylor SA, James M, Groot N, MacFarlane H. A novel gene containing a trinucleotide repeat that is expanded and unstable on Huntington’s disease chromosomes. Cell. 1993;72:971–83. 10.1016/0092-8674(93)90585-e 8458085

[137] Ryu H, Lee J, Hagerty SW, Soh BY, McAlpin SE, Cormier KA, et al. ESET/SETDB1 gene expression and histone H3 (K9) trimethylation in Huntington’s disease. Proc Natl Acad Sci USA. 2006;103:19176–81. 17142323 10.1073/pnas.0606373103 PMC1748195

[138] Lee J, Hwang YJ, Kim Y, Lee MY, Hyeon SJ, Lee S, et al. Remodeling of heterochromatin structure slows neuropathological progression and prolongs survival in an animal model of Huntington’s disease. Acta Neuropathol. 2017;134:729–48. 28593442 10.1007/s00401-017-1732-8

[139] Dong X, Tsuji J, Labadorf A, Roussos P, Chen JF, Myers RH, et al. The role of H3K4me3 in transcriptional regulation is altered in Huntington’s disease. PloS ONE. 2015;10:e0144398. 26636336 10.1371/journal.pone.0144398 PMC4670094

[140] Nucifora FC Jr., Sasaki M, Peters MF, Huang H, Cooper JK, Yamada M, et al. Interference by huntingtin and atrophin-1 with cbp-mediated transcription leading to cellular toxicity. Science. 2001;291:2423–8. 11264541 10.1126/science.1056784

[141] Developmental alterations in Huntington’s disease neural cells and pharmacological rescue in cells and mice. Nat Neurosci. 2017;20:648–60. 10.1038/nn.4532 PMC5610046 28319609

[142] Narayan P, Reid S, Scotter EL, McGregor AL, Mehrabi NF, Singh-Bains MK, et al. Inconsistencies in histone acetylation patterns among different HD model systems and HD post-mortem brains. Neurobiol Dis. 2020;146:105092. 32979507 10.1016/j.nbd.2020.105092

[143] Hyeon JW, Kim AH, Yano H. Epigenetic regulation in Huntington’s disease. Neurochem Int. 2021;148:105074. 34038804 10.1016/j.neuint.2021.105074 PMC9110274

[144] Lu AT, Narayan P, Grant MJ, Langfelder P, Wang N, Kwak S, et al. DNA methylation study of Huntington’s disease and motor progression in patients and in animal models. Nat Commun. 2020;11:4529. 32913184 10.1038/s41467-020-18255-5 PMC7484780

[145] Fernández-Nogales M, Cabrera JR, Santos-Galindo M, Hoozemans JJ, Ferrer I, Rozemuller AJ, et al. Huntington’s disease is a four-repeat tauopathy with tau nuclear rods. Nat Med. 2014;20:881–5. 25038828 10.1038/nm.3617

[146] Blum D, Herrera F, Francelle L, Mendes T, Basquin M, Obriot H, et al. Mutant huntingtin alters Tau phosphorylation and subcellular distribution. Hum Mol Genet. 2015;24:76–85. 25143394 10.1093/hmg/ddu421

[147] Gratuze M, Noël A, Julien C, Cisbani G, Milot-Rousseau P, Morin F, et al. Tau hyperphosphorylation and deregulation of calcineurin in mouse models of Huntington’s disease. Hum Mol Genet. 2015;24:86–99. 25205109 10.1093/hmg/ddu456

[148] Feldman EL, Goutman SA, Petri S, Mazzini L, Savelieff MG, Shaw PJ, et al. Amyotrophic lateral sclerosis. Lancet. 2022;400:1363–80. 36116464 10.1016/S0140-6736(22)01272-7 PMC10089700

[149] Boeve BF, Boxer AL, Kumfor F, Pijnenburg Y, Rohrer JD. Advances and controversies in frontotemporal dementia: diagnosis, biomarkers, and therapeutic considerations. Lancet Neurol. 2022;21:258–72. 35182511 10.1016/S1474-4422(21)00341-0

[150] Lomen-Hoerth C. Clinical phenomenology and neuroimaging correlates in ALS-FTD. J Mol Neurosci. 2011;45:656–62. 21971978 10.1007/s12031-011-9636-x

[151] Mackenzie IR, Rademakers R, Neumann M. TDP-43 and FUS in amyotrophic lateral sclerosis and frontotemporal dementia. Lancet Neurol. 2010;9:995–1007. 20864052 10.1016/S1474-4422(10)70195-2

[152] Fecto F, Siddique T. Making connections: pathology and genetics link amyotrophic lateral sclerosis with frontotemporal lobe dementia. J Mol Neurosci. 2011;45:663–75. 21901496 10.1007/s12031-011-9637-9

[153] Abramzon YA, Fratta P, Traynor BJ, Chia R. The overlapping genetics of amyotrophic lateral sclerosis and frontotemporal dementia. Front Neurosci. 2020;14:42. 32116499 10.3389/fnins.2020.00042 PMC7012787

[154] Niccoli T, Partridge L, Isaacs AM. Ageing as a risk factor for ALS/FTD. Hum Mol Genet. 2017;26:R105–r113. 28977441 10.1093/hmg/ddx247

[155] Johnson SA, Fang T, De Marchi F, Neel D, Van Weehaeghe D, Berry JD, et al. Pharmacotherapy for amyotrophic lateral sclerosis: a review of approved and upcoming agents. Drugs. 2022;82:1367–88. 36121612 10.1007/s40265-022-01769-1

[156] Fisher RMA, Torrente MP. Histone post-translational modification and heterochromatin alterations in neurodegeneration: revealing novel disease pathways and potential therapeutics. Front Mol Neurosci. 2024;17:1456052. 39346681 10.3389/fnmol.2024.1456052 PMC11427407

[157] DeJesus-Hernandez M, Mackenzie IR, Boeve BF, Boxer AL, Baker M, Rutherford NJ, et al. Expanded GGGGCC hexanucleotide repeat in noncoding region of C9ORF72 causes chromosome 9p-linked FTD and ALS. Neuron. 2011;72:245–56. 21944778 10.1016/j.neuron.2011.09.011 PMC3202986

[158] Belzil VV, Bauer PO, Prudencio M, Gendron TF, Stetler CT, Yan IK, et al. Reduced C9orf72 gene expression in c9FTD/ALS is caused by histone trimethylation, an epigenetic event detectable in blood. Acta Neuropathol. 2013;126:895–905. 24166615 10.1007/s00401-013-1199-1 PMC3830740

[159] Esanov R, Cabrera GT, Andrade NS, Gendron TF, Brown RH Jr, Benatar M, et al. A C9ORF72 BAC mouse model recapitulates key epigenetic perturbations of ALS/FTD. Mol Neurodegener. 2017;12:46. 28606110 10.1186/s13024-017-0185-9 PMC5468954

[160] Jury N, Abarzua S, Diaz I, Guerra MV, Ampuero E, Cubillos P, et al. Widespread loss of the silencing epigenetic mark H3K9me3 in astrocytes and neurons along with hippocampal-dependent cognitive impairment in C9orf72 BAC transgenic mice. Clin Epigenetics. 2020;12:32. 32070418 10.1186/s13148-020-0816-9 PMC7029485

[161] Li J, Jaiswal MK, Chien JF, Kozlenkov A, Jung J, Zhou P, et al. Divergent single cell transcriptome and epigenome alterations in ALS and FTD patients with C9orf72 mutation. Nat Commun. 2023;14:5714. 37714849 10.1038/s41467-023-41033-y PMC10504300

[162] Liu Y, Huang Z, Liu H, Ji Z, Arora A, Cai D, et al. DNA-initiated epigenetic cascades driven by C9orf72 hexanucleotide repeat. Neuron. 2023;111:1205–1221.e1209. 36822200 10.1016/j.neuron.2023.01.022 PMC10121948

[163] Chen K, Bennett SA, Rana N, Yousuf H, Said M, Taaseen S, et al. Neurodegenerative disease proteinopathies are connected to distinct histone post-translational modification landscapes. ACS Chem Neurosci. 2018;9:838–48. 29243911 10.1021/acschemneuro.7b00297 PMC5906139

[164] Wu CC, Jin LW, Wang IF, Wei WY, Ho PC, Liu YC, et al. HDAC1 dysregulation induces aberrant cell cycle and DNA damage in progress of TDP-43 proteinopathies. EMBO Mol Med. 2020;12:e10622. 32449313 10.15252/emmm.201910622 PMC7278561

[165] Schwartz JC, Podell ER, Han SS, Berry JD, Eggan KC, Cech TR. FUS is sequestered in nuclear aggregates in ALS patient fibroblasts. Mol Biol Cell. 2014;25:2571–8. 25009283 10.1091/mbc.E14-05-1007 PMC4148247

[166] Tibshirani M, Tradewell ML, Mattina KR, Minotti S, Yang W, Zhou H, et al. Cytoplasmic sequestration of FUS/TLS associated with ALS alters histone marks through loss of nuclear protein arginine methyltransferase 1. Hum Mol Genet. 2015;24:773–86. 25274782 10.1093/hmg/ddu494 PMC4291251

[167] Rossaert E, Pollari E, Jaspers T, Van Helleputte L, Jarpe M, Van Damme P, et al. Restoration of histone acetylation ameliorates disease and metabolic abnormalities in a FUS mouse model. Acta Neuropathol Commun. 2019;7:107. 31277703 10.1186/s40478-019-0750-2 PMC6612190

[168] Arenas A, Chen J, Kuang L, Barnett KR, Kasarskis EJ, Gal J, et al. Lysine acetylation regulates the RNA binding, subcellular localization and inclusion formation of FUS. Hum Mol Genet. 2020;29:2684–97. 32691043 10.1093/hmg/ddaa159 PMC7530527

[169] Thompson AJ, Baranzini SE, Geurts J, Hemmer B, Ciccarelli O. Multiple sclerosis. Lancet. 2018;391:1622–36. 29576504 10.1016/S0140-6736(18)30481-1

[170] Correale J, Gaitán MI, Ysrraelit MC, Fiol MP. Progressive multiple sclerosis: from pathogenic mechanisms to treatment. Brain. 2017;140:527–46. 27794524 10.1093/brain/aww258

[171] Ascherio A, Munger KL, Lünemann JD. The initiation and prevention of multiple sclerosis. Nat Rev Neurol. 2012;8:602–12. 23045241 10.1038/nrneurol.2012.198 PMC4467212

[172] He H, Hu Z, Xiao H, Zhou F, Yang B. The tale of histone modifications and its role in multiple sclerosis. Hum Genom. 2018;12:31. 10.1186/s40246-018-0163-5 PMC6013900 29933755

[173] Seder RA, Ahmed R. Similarities and differences in CD4+ and CD8+ effector and memory T cell generation. Nat Immunol. 2003;4:835–42. 12942084 10.1038/ni969

[174] Larochelle C, Alvarez JI, Prat A. How do immune cells overcome the blood-brain barrier in multiple sclerosis? FEBS Lett. 2011;585:3770–80. 21550344 10.1016/j.febslet.2011.04.066

[175] Muraille E, Leo O, Moser M. TH1/TH2 paradigm extended: macrophage polarization as an unappreciated pathogen-driven escape mechanism? Front Immunol. 2014;5:603. 25505468 10.3389/fimmu.2014.00603 PMC4244692

[176] Wang L, Tao R, Hancock WW. Using histone deacetylase inhibitors to enhance Foxp3(+) regulatory T-cell function and induce allograft tolerance. Immunol Cell Biol. 2009;87:195–202. 19172156 10.1038/icb.2008.106

[177] Sun L, Telles E, Karl M, Cheng F, Luetteke N, Sotomayor EM, et al. Loss of HDAC11 ameliorates clinical symptoms in a multiple sclerosis mouse model. Life Sci Alliance. 2018;1:e201800039. 30456376 10.26508/lsa.201800039 PMC6238389

[178] Zhang Z, Zhang ZY, Wu Y, Schluesener HJ. Valproic acid ameliorates inflammation in experimental autoimmune encephalomyelitis rats. Neuroscience. 2012;221:140–50. 22800566 10.1016/j.neuroscience.2012.07.013

[179] Ge Z, Da Y, Xue Z, Zhang K, Zhuang H, Peng M, et al. Vorinostat, a histone deacetylase inhibitor, suppresses dendritic cell function and ameliorates experimental autoimmune encephalomyelitis. Exp Neurol. 2013;241:56–66. 23261766 10.1016/j.expneurol.2012.12.006

[180] Liu Z, Cao W, Xu L, Chen X, Zhan Y, Yang Q, et al. The histone H3 lysine-27 demethylase Jmjd3 plays a critical role in specific regulation of Th17 cell differentiation. J Mol Cell Biol. 2015;7:505–16. 25840993 10.1093/jmcb/mjv022

[181] Marin-Husstege M, Muggironi M, Liu A, Casaccia-Bonnefil P. Histone deacetylase activity is necessary for oligodendrocyte lineage progression. J Neurosci. 2002;22:10333–45. 12451133 10.1523/JNEUROSCI.22-23-10333.2002 PMC6758756

[182] Shen S, Sandoval J, Swiss VA, Li J, Dupree J, Franklin RJ, et al. Age-dependent epigenetic control of differentiation inhibitors is critical for remyelination efficiency. Nat Neurosci. 2008;11:1024–34. 19160500 10.1038/nn.2172 PMC2656679

[183] Pedre X, Mastronardi F, Bruck W, López-Rodas G, Kuhlmann T, Casaccia P. Changed histone acetylation patterns in normal-appearing white matter and early multiple sclerosis lesions. J Neurosci. 2011;31:3435–45. 21368055 10.1523/JNEUROSCI.4507-10.2011 PMC3081530

[184] Nativio R, Donahue G, Berson A, Lan Y, Amlie-Wolf A, Tuzer F, et al. Dysregulation of the epigenetic landscape of normal aging in Alzheimer’s disease. Nat Neurosci. 2018;21:497–505. 29507413 10.1038/s41593-018-0101-9 PMC6124498

[185] Tang B, Dean B, Thomas EA. Disease- and age-related changes in histone acetylation at gene promoters in psychiatric disorders. Transl Psychiatry. 2011;1:e64. 22832356 10.1038/tp.2011.61 PMC3305989

[186] Persico G, Casciaro F, Amatori S, Rusin M, Cantatore F, Perna A, et al. Histone H3 Lysine 4 and 27 trimethylation landscape of human Alzheimer’s Disease. Cells. 2022;11:734. 10.3390/cells11040734 PMC8870338 35203383

[187] Luchsinger JA, Perez T, Chang H, Mehta P, Steffener J, Pradabhan G, et al. Metformin in amnestic mild cognitive impairment: results of a pilot randomized placebo controlled clinical trial. J Alzheimer’s Dis. 2016;51:501–14. 26890736 10.3233/JAD-150493 PMC5079271

[188] Fernando W, Martins IJ, Morici M, Bharadwaj P, Rainey-Smith SR, Lim WLF, et al. Sodium butyrate reduces brain Amyloid-β levels and improves cognitive memory performance in an Alzheimer’s disease transgenic mouse model at an early disease stage. J Alzheimer’s Dis. 2020;74:91–9. 31958090 10.3233/JAD-190120

[189] Wang F, Zhang Z, Sun Y, Yang L, Guo T, Pan Y, et al. Bmal1 mediates the neuroprotective effect of sodium butyrate in a mouse model of Parkinson’s disease. J South Med Univ. 2024;44:876–84. 10.12122/j.issn.1673-4254.2024.05.09 PMC11166718 38862445

[190] Xuan AG, Pan XB, Wei P, Ji WD, Zhang WJ, Liu JH, et al. Valproic acid alleviates memory deficits and attenuates amyloid-β deposition in transgenic mouse model of Alzheimer’s disease. Mol Neurobiol. 2015;51:300–12. 24854198 10.1007/s12035-014-8751-4

[191] Ximenes JC, Neves KR, Leal LK, do Carmo MR, Brito GA, Naffah-Mazzacoratti Mda G, et al. Valproic acid neuroprotection in the 6-OHDA model of parkinson’s disease is possibly related to its anti-inflammatory and HDAC inhibitory properties. J Neurodegener Dis. 2015;2015:313702. 26317011 10.1155/2015/313702 PMC4437346

[192] Su Q, Li T, He PF, Lu XC, Yu Q, Gao QC, et al. Trichostatin A ameliorates Alzheimer’s disease-related pathology and cognitive deficits by increasing albumin expression and Aβ clearance in APP/PS1 mice. Alzheimer’s Res Ther. 2021;13:7. 33397436 10.1186/s13195-020-00746-8 PMC7784383

[193] Suo H, Wang P, Tong J, Cai L, Liu J, Huang D, et al. NRSF is an essential mediator for the neuroprotection of trichostatin A in the MPTP mouse model of Parkinson’s disease. Neuropharmacology. 2015;99:67–78. 26188143 10.1016/j.neuropharm.2015.07.015

[194] Athaide Rocha KM, Machado FR, Poetini M, Giacomeli R, Boeira SP, Jesse CR, et al. Assessment of suberoylanilide hydroxamic acid on a Alzheimer’s disease model induced by β-amyloid((1-42)) in aged female mice: neuromodulatory and epigenetic effect. Chem-Biol Interact. 2023;375:110429. 36870467 10.1016/j.cbi.2023.110429

[195] Chen SH, Wu HM, Ossola B, Schendzielorz N, Wilson BC, Chu CH, et al. Suberoylanilide hydroxamic acid, a histone deacetylase inhibitor, protects dopaminergic neurons from neurotoxin-induced damage. Br J Pharmacol. 2012;165:494–505. 21726209 10.1111/j.1476-5381.2011.01575.x PMC3268201

[196] Hockly E, Richon VM, Woodman B, Smith DL, Zhou X, Rosa E, et al. Suberoylanilide hydroxamic acid, a histone deacetylase inhibitor, ameliorates motor deficits in a mouse model of Huntington’s disease. Proc Natl Acad Sci USA. 2003;100:2041–6. 12576549 10.1073/pnas.0437870100 PMC149955

[197] Moussa C, Hebron M, Huang X, Ahn J, Rissman RA, Aisen PS, et al. Resveratrol regulates neuro-inflammation and induces adaptive immunity in Alzheimer’s disease. J Neuroinflamm. 2017;14:1. 10.1186/s12974-016-0779-0 PMC5234138 28086917

[198] Griñán-Ferré C, Marsal-García L, Bellver-Sanchis A, Kondengaden SM, Turga RC, Vázquez S, et al. Pharmacological inhibition of G9a/GLP restores cognition and reduces oxidative stress, neuroinflammation and β-Amyloid plaques in an early-onset Alzheimer’s disease mouse model. Aging. 2019;11:11591–608. 31804189 10.18632/aging.102558 PMC6932909

[199] Cheng FF, Liu YL, Du J, Lin JT. Metformin’s mechanisms in attenuating hallmarks of aging and age-related disease. Aging Dis. 2022;13:970–86. 35855344 10.14336/AD.2021.1213 PMC9286921

[200] Lu M, Su C, Qiao C, Bian Y, Ding J, Hu G. Metformin prevents dopaminergic neuron death in MPTP/P-induced mouse model of Parkinson’s disease via autophagy and mitochondrial ROS clearance. Int J Neuropsychopharmacol. 2016;19:pyw047. 10.1093/ijnp/pyw047 PMC5043649 27207919

[201] Lu XY, Huang S, Chen QB, Zhang D, Li W, Ao R, et al. Metformin ameliorates Aβ pathology by insulin-degrading enzyme in a transgenic mouse model of Alzheimer’s disease. Oxid Med Cell Longev. 2020;2020:2315106. 32377293 10.1155/2020/2315106 PMC7191377

[202] Thellung S, Corsaro A, Nizzari M, Barbieri F, Florio T. Autophagy activator drugs: a new opportunity in neuroprotection from misfolded protein toxicity. Int J Mol Sci. 2019;20:901. 10.3390/ijms20040901 PMC6412775 30791416

[203] Paniri A, Hosseini MM, Akhavan-Niaki H. Alzheimer’s disease-related epigenetic changes: novel therapeutic targets. Mol Neurobiol. 2024;61:1282–317. 37700216 10.1007/s12035-023-03626-y

[204] Gupta R, Ambasta RK, Kumar P. Pharmacological intervention of histone deacetylase enzymes in the neurodegenerative disorders. Life Sci. 2020;243:117278. 31926248 10.1016/j.lfs.2020.117278

[205] Bondarev AD, Attwood MM, Jonsson J, Chubarev VN, Tarasov VV, Schiöth HB. Recent developments of HDAC inhibitors: emerging indications and novel molecules. Br J Clin Pharmacol. 2021;87:4577–97. 33971031 10.1111/bcp.14889

[206] d’Ydewalle C, Krishnan J, Chiheb DM, Van Damme P, Irobi J, Kozikowski AP, et al. HDAC6 inhibitors reverse axonal loss in a mouse model of mutant HSPB1-induced Charcot-Marie-Tooth disease. Nat Med. 2011;17:968–74. 21785432 10.1038/nm.2396

[207] Pulya S, Amin SA, Adhikari N, Biswas S, Jha T, Ghosh B. HDAC6 as privileged target in drug discovery: a perspective. Pharmacol Res. 2021;163:105274. 33171304 10.1016/j.phrs.2020.105274

[208] Collins LM, Adriaanse LJ, Theratile SD, Hegarty SV, Sullivan AM, O’Keeffe GW. Class-IIa histone deacetylase inhibition promotes the growth of neural processes and protects them against neurotoxic insult. Mol Neurobiol. 2015;51:1432–42. 25065734 10.1007/s12035-014-8820-8

[209] Fu J, An L. Histone methylation, energy metabolism, and Alzheimer’s disease. Aging Dis. 2024;16:2831–58. 39656495 10.14336/AD.2024.0899 PMC12339095

[210] Monteiro-Junior RS, Cevada T, Oliveira BR, Lattari E, Portugal EM, Carvalho A, et al. We need to move more: neurobiological hypotheses of physical exercise as a treatment for Parkinson’s disease. Med hypotheses. 2015;85:537–41. 26209418 10.1016/j.mehy.2015.07.011

[211] Gomez-Pinilla F, Thapak P. Exercise epigenetics is fueled by cell bioenergetics: supporting role on brain plasticity and cognition. Free Radic Biol Med. 2024;220:43–55. 38677488 10.1016/j.freeradbiomed.2024.04.237 PMC11144461

[212] Ravussin E, Redman LM, Rochon J, Das SK, Fontana L, Kraus WE, et al. A 2-year randomized controlled trial of human caloric restriction: feasibility and effects on predictors of health span and longevity. J Gerontol Ser A Biomed Sci Med Sci. 2015;70:1097–104. 10.1093/gerona/glv057 PMC4841173 26187233

[213] Fortier M, Castellano CA, St-Pierre V, Myette-Côté É, Langlois F, Roy M, et al. A ketogenic drink improves cognition in mild cognitive impairment: results of a 6-month RCT. Alzheimer’s Dement. 2021;17:543–52. 33103819 10.1002/alz.12206 PMC8048678

[214] Zhou T, Cheng X, He Y, Xie Y, Xu F, Xu Y, et al. Function and mechanism of histone β-hydroxybutyrylation in health and disease. Front Immunol. 2022;13:981285. 36172354 10.3389/fimmu.2022.981285 PMC9511043

[215] Jang J, Kim SR, Lee JE, Lee S, Son HJ, Choe W, et al. Molecular mechanisms of neuroprotection by ketone bodies and ketogenic diet in cerebral ischemia and neurodegenerative diseases. Int J Mol Sci. 2023;25:124. 10.3390/ijms25010124 PMC10779133 38203294

[216] Liu X, Liu Z, Wu Z, Ren J, Fan Y, Sun L, et al. Resurrection of endogenous retroviruses during aging reinforces senescence. Cell. 2023;186:287–304.e226. 36610399 10.1016/j.cell.2022.12.017

[217] Yang Z, Gong M, Jian T, Li J, Yang C, Ma Q, et al. Engrafted glial progenitor cells yield long-term integration and sensory improvement in aged mice. Stem Cell Res Ther. 2022;13:285. 35765112 10.1186/s13287-022-02959-0 PMC9241208

[218] Antón-Fernández A, Ruiz de Alegría Á, Mariscal-Casero A, Roldán-Lázaro M, Peinado-Cauchola R, Ávila J, et al. Partial reprogramming by cyclical overexpression of Yamanaka factors improves pathological phenotypes of tauopathy mouse model of human Alzheimer’s disease. Prog Neurobiol. 2025;247:102743. 40021076 10.1016/j.pneurobio.2025.102743

[219] Ocampo A, Reddy P, Martinez-Redondo P, Platero-Luengo A, Hatanaka F, Hishida T, et al. In vivo amelioration of age-associated hallmarks by partial reprogramming. Cell. 2016;167:1719–33.e1712. 27984723 10.1016/j.cell.2016.11.052 PMC5679279

[220] Wu Z, Qu J, Zhang W, Liu GH. Stress, epigenetics, and aging: unraveling the intricate crosstalk. Mol Cell. 2024;84:34–54. 37963471 10.1016/j.molcel.2023.10.006

[221] Sanna S, Esposito S, Masala A, Sini P, Nieddu G, Galioto M, et al. HDAC1 inhibition ameliorates TDP-43-induced cell death in vitro and in vivo. Cell Death Dis. 2020;11:369. 32409664 10.1038/s41419-020-2580-3 PMC7224392

[222] Li Y, Zhao Y, Li X, Zhai L, Zheng H, Yan Y, et al. Biological and therapeutic role of LSD1 in Alzheimer’s diseases. Front Pharmacol. 2022;13:1020556. 36386192 10.3389/fphar.2022.1020556 PMC9640401

